# Mental Health Support Workers Perspectives on Barriers to and Facilitators of the Effective Delivery of their Roles: A Systematic Review and Meta-aggregation

**DOI:** 10.1007/s10597-025-01490-9

**Published:** 2025-07-24

**Authors:** Martha Njuguna, Irene Ngune, Yvonne Middlewick

**Affiliations:** School of Nursing and Midwifery, Edith Cowan University, 270 Joondalup Drive, Joondalup, WA 6027 Australia

**Keywords:** Mental health support worker, Care worker, Mental health, Mental illness, Mental health recovery, Mental disorder

## Abstract

Mental health support workers (MHSWs) are an important workforce within the mental health care system. Research shows that support services delivered by MHSWs are effective at improving the outcomes of individuals with mental illness. However, there is also literature suggesting that this group of workers are facing many challenges in their roles. This qualitative systematic review sought to explore, analyze and synthesize available primary research literature on the experiences of MHSWs with the aim of identifying the barriers to and facilitators of effective delivery of their roles. An electronic search of Medline, CINAHL, PsycINFO, and Web of Science was conducted for studies from database inception to March 28, 2025. Additional search was conducted via Google Scholar and in the reference lists of the included articles. The Joanna Briggs Institute (JBI) Critical Appraisal Checklist for Qualitative Research was used to assess the methodological quality of included articles. Fourteen primary research articles met the eligibility criteria and were included in the review. Data synthesis from these articles generated 116 findings and 20 categories, which were aggregated into four synthesized findings: a supportive work environment, service user care barriers, professional and role-related barriers, and operational and support barriers. The review highlights the importance of a supportive work environment in the delivery of support work roles. However, several barriers were also identified and recommendations that can be used to inform practice and policies of mental health support work provided. If addressed, the identified barriers can be translated into facilitators that would enhance the provision of quality care to people with mental illness and enhance the overall job satisfaction of MHSWs.

**Supplementary Information:**

The online version contains supplementary material available at 10.1007/s10597-025-01490-9.

## Introduction

Mental illnesses are a leading cause of disability and disease burden, affecting approximately 10% of the global population (Patel & Saxena, 2014). In Australia, 1 in 5 people experience mental health issues each year, with 5% estimated to have a severe mental illness (Australian Institute for Health and Welfare (AIHW), 2022) a clinically diagnosable mental, behavioral or emotional disorder that is episodic, recurrent, or persistent and causes disruption in psychosocial and occupational functioning (Baker et al., 2018).

The impact is experienced not only by individuals with lived and living experience but also by their families and society (AIHW, 2022). This is further compounded by the cost of hospital treatment, higher unemployment rates, increased social exclusion and reduced life expectancy due to long-term conditions, resulting in a significant economic impact. In Australia, the total economic cost of mental illness is estimated to be $220 billion per year (Productivity Commission., 2020).

To reduce the impact of mental illnesses, government policies around the world have focused on making healthcare services accessible (Barry & Huskamp, 2011). However, accessing mental health services remains challenging due to factors such as high costs, limited availability of treatment, and stigma. These barriers are postulated to be due to a shortage of mental health professionals, inadequate mental health services and limited funding (Becker & Kleinman, 2013). The World Health Organization (WHO, 2022) recommends enhancing services and expanding the mental health workforce by introducing new roles, such as mental health support workers (MHSWs). According to Kazdin (2017), new roles can increase the accessibility of healthcare services. This strategy is cost-effective (Kazdin & Rabbitt, 2013) and can potentially reduce the global mental health care disparities for underserved populations (Barnett et al., 2018). This initiative aligns with United Nations Sustainable Development Goal 3 (SDG 3), which aims to reduce health inequalities by improving access to health services for vulnerable populations, including individuals living with severe mental illness (United Nations, n.d.).

The role of the MHSW focuses on fostering independence by providing emotional and practical support to service users (Manthorpe et al., 2010). Support includes services such as assisting service users with activities of daily living, decision making, community and social events participation, managing medications as well as learning new skills (Andersson, 2016; Kirkpatrick & Byrne, 2011; Kowlessar & Corbett, 2009; Muir et al., 2010; Russinova et al., 2011). The MHSW role is complementary to and has the potential to further enhance the work of mental health professionals, thereby offering an opportunity to further assist the person living with a mental illness. A systematic review by Hoeft et al. (2018) found that redistributing tasks from highly trained clinicians to less highly trained workers in rural and low-resource settings was an efficient use of limited resources. The development of the MHSW role may, in part, be a response to limited access to mental health professionals due to workforce shortages (Commonwealth of Australia, 2023). However, beyond addressing workforce gaps, this role also presents a valuable opportunity to enhance service delivery through expanded access to community-based, recovery-oriented support. The training of MHSWs varies across jurisdictions depending on local health needs (Rifkin, 2017; WHO, 2022). Despite these variations, evidence suggests that the services provided by MHSWs are effective at increasing service users’ satisfaction and improving their mental health outcomes (Barnett et al., 2018; Siskind et al., 2012).

The role of mental health support workers (MHSWs) should be framed within a recovery-oriented approach, alongside that of other mental health professionals, as they collectively contribute to supporting individuals in leading meaningful and self-directed lives. Recovery-oriented practice is underpinned by the belief that people living with mental illness can be empowered to live a satisfying life, with or without ongoing symptoms (Davidson et al., 2007; Slade, 2009). This has been shaped by the experiences of people with lived experience and what they have found helpful during their recovery process (Brekke et al., 2018; Byrne et al., 2013). This approach shifts the focus of care from symptom reduction alone to empowering people living with a mental illness to live, work, learn and engage in their communities (Davidson et al., 2016). Recovery-oriented practice emphasizes person-centred care, autonomy, hope and community participation, with support being aligned to the goals and values of the individual receiving the services. Slade (2009) highlights that recovery is not a treatment outcome, but a deeply personal process shaped by the individual's own goals, values and relationships. According to Manthorpe et al. (2010) MHSWs play an important role in delivering personalized, relationship-based support services that align with the values of recovery-oriented practice.

The mobilization of support workers'roles in mental health services has increased significantly in the past few years. However, research suggests that these workers face a number of work-related challenges. For example, Saari et al. (2017) identified barriers such as poor communication, lack of standardized training, inadequate support, and unclear roles. Similarly, Belling et al. (2011) highlighted issues such as blurred professional boundaries and insufficient training as key barriers to the continuity of care in community mental health teams. A review by Hoeft et al. (2018) also identified several challenges associated with redistributing tasks to a less highly trained workforce, including concerns around professional boundaries, confidentiality, burnout, and staff turnover. These findings highlight the challenges that MHSWs face in their roles, including the complexity of integrating their work within the existing mental health systems. Capturing the perspectives and understanding the barriers and facilitators MHSWs face in their roles, which have not yet been systematically reviewed, could support the effective development of their roles to enhance the quality of care they provide and improve the mental health outcomes of service users.

### Aim

This systematic review aimed to identify the barriers to and facilitators of the effective delivery of MHSWs roles as perceived by MHSWs.

## Methodology

The Joanna Briggs Institute (JBI) was the first to develop the meta-aggregation approach with the aim of providing a structured, systematic approach to qualitative evidence (Lockwood et al., 2015, 2020). The meta-aggregation process is a step-by-step systematic review methodology sensitive to the traditions and contextual nature of qualitative research (Lockwood et al., 2015). The qualitative findings from the included articles are integrated to form a synthesized finding, forming the basis for recommendations to guide practice and policy development (Lockwood et al., 2015).

This approach was chosen over other qualitative methodologies, such as meta-ethnography, which focuses on generating new theoretical insights, because the primary aim of this review was to identify gaps and produce actionable findings to inform policy and practice, aligning with the core purpose of the JBI meta-aggregation approach. The review protocol was registered in PROSPERO on 30 October 2023, registration number CRD42023473835.

### Inclusion and Exclusion Criteria

Studies were included if they:1) used a qualitative research design; mixed-methods studies were included if the qualitative part was separate from the quantitative part of the study; 2) reported the experiences and perspectives of MHSWs providing support services to adults with mental illness; 3) were full–text, primary research articles, including theses published in English; and 4) reported on support services provided in a community setting, including but not limited to care provided in group homes, supported accommodations and independent living and home-based care. Outpatient mental health clinics were excluded, as the focus of this review was non-clinical, psychosocial support provided in residential or community-integrated environments, which were considered as the place where the person lives. For studies with samples of MHSWs, peer support workers, service users, health professionals, and other key stakeholders, only the experiences and perspectives of MHSWs were included in the review. The time frame for the included articles was not restricted to identify available literature exploring MHSWs experiences.

### Search Strategy

An initial search was developed in MEDLINE to test the search terms and validated in collaboration with a university librarian. This was followed by a comprehensive database search using MEDLINE, the Cumulative Index of Nursing and Allied Health (CINAHL), PsycINFO, and Web of Science for published studies, grey literature sources for unpublished studies and a reference list search of included articles. The keywords included “support worker”, “health care assistant”, “health care aide”, “home health aide”, “lay health worker”, “community health aide”,"mental disorder”, “mental illness”, “mental disorder”, “severe mental illness”, “community mental health services” and “recovery”.

The search strategy was tailored for each database using keywords, synonyms, and index terms. An initial search conducted on 6 September 2023 yielded 492 articles. A second search was performed on 28 March 2025 to capture any newly published literature, and an additional 55 articles were identified. Additionally, a supplementary internet search was conducted via Google Scholar on 20 October 2023 and on 28 March 2025 using the terms “support worker” or “support workers” and “mental health” or “mental illness”, not “peer support workers”. Fewer search terms were used due to the limited search functionalities of Google Scholar compared to databases, and to maximize the retrieval of relevant articles. The first 100 entries were reviewed. The reference lists of the included articles were also searched.

**Fig. 1:**
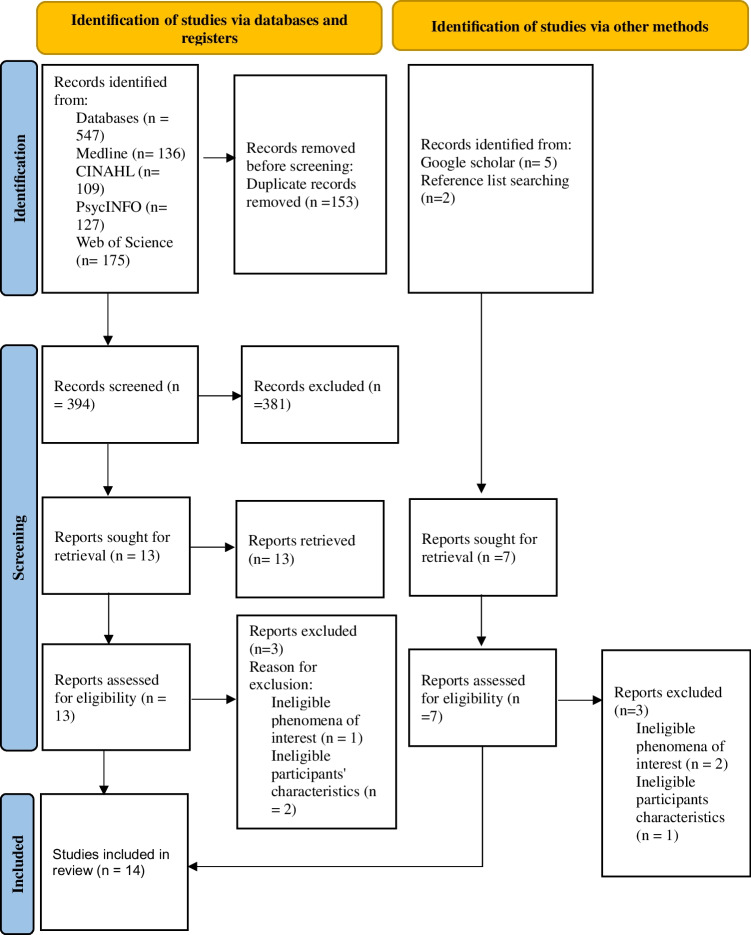
Preferred reporting items for systematic reviews and meta-analysis (PRISMA) flow diagram

The database searches (Appendix 1) yielded 547 articles, including 153 duplicates, which were removed before screening (Fig. 1). The Google search and reference list search yielded seven articles.

### Assessment of Methodological Quality

Critical appraisal of the included articles is an important part of the meta-aggregation approach, which aims to generate findings that can inform healthcare policies and practices (Lockwood et al., 2020). This review used the JBI Critical Appraisal Checklist for Qualitative Research (Lockwood et al., 2020), designed for health-related studies (Appendix 2). Two reviewers (MN and YM) independently conducted the appraisal, and conflicts were resolved through discussion. For example, when comparing the outcomes of the appraisal of one article (Wilberforce et al., 2017), there was a disagreement regarding the JBI Critical Appraisal Checklist of qualitative studies question 6: “Is there a statement locating the researcher culturally or theoretically?” One reviewer rated this as No, while the other rated it as Unclear. After discussion and further examination of the article by both reviewers, they agreed that there was no clear statement locating the researcher culturally or theoretically, and the final rating was revised to No. All conflicts were resolved using this approach. The quality appraisal results were recorded using JBI SUMARI, and no articles were excluded based on the quality assessment.

### Data Extraction

A standardized data extraction tool from the JBI Qualitative Assessment and Review Instrument (JBI-QARI) located in JBI SUMARI (Appendix 3) was used to extract the data. All steps were completed by one reviewer (MN) and verified by a second reviewer (YM). Data extraction followed the phases of the meta-aggregative approach (Lockwood et al., 2015). In phase one, information illustrating the characteristics of the included studies was extracted. In the second phase, which is also the first step of data synthesis, specific study findings relevant to the review question were extracted through multiple readings of the studies'findings. Findings were included if they were i) a verbatim excerpt of the author’s study description or an interpretation of the qualitative data in their study, and ii) accompanied by supporting illustrations such as direct quotations from participants.

### Data Synthesis

Data synthesis was conducted in three steps: 1) Findings were extracted and assigned a level of credibility based on the congruency between the findings and their accompanying illustration. The illustration of the finding was a quotation or a collection of quotations from the paper. The Findings were classified as unequivocal (U) if fully supported by the illustration, Credible (C) if the association between the finding and the illustration was not clear and unsupported (NS) if no connection between the finding and the illustration was identified (Appendix 5) (Lockwood et al., 2015). Unsupported findings were excluded from the data synthesis. 2) The unequivocal and credible findings were grouped into categories of two or more findings with similar meanings or concepts (Lockwood et al., 2015). Each category was allocated a label reflecting the overarching meaning of the included findings. 3) Categories with similar meanings were merged to generate overarching assertions, which formed the basis for recommendations for healthcare practice and policy (Lockwood et al., 2015).

## Findings

### Study Selection

All the articles retrieved from the databases were saved into EndNote version 20, and duplicates were removed. The remaining articles were imported into Rayyan for screening. Two reviewers (MN and IN) independently screened the titles and abstracts of 394 articles and excluded 381 articles based on the inclusion/exclusion criteria. Conflicts were resolved through discussion or with a third reviewer (YM). The remaining articles, grey literature and articles identified through reference list search were imported into the JBI System for the Unified Management, Assessment and Review of Information (JBI SUMARI) (Munn et al., 2019) for full text review. MN and YM independently reviewed twenty articles, and six (Appendix 4) were excluded due to ineligible participant characteristics and phenomena of interest. Fourteen articles met the inclusion criteria and were included for data extraction and synthesis. The PRISMA flow diagram (Fig. 1) illustrates the process of identification, screening, and inclusion.

### Study Quality Assessment

The included papers demonstrated high methodological quality in several key areas, such as congruity between research methodology and the research question or objectives (Q2), data collection methods (Q3), data representation and analysis (Q4), and result interpretation (Q5). Study participants were adequately represented (Q8), and ethical approval was obtained (Q9) for all studies. However, some studies did not demonstrate congruency between the methodology and the stated philosophical perspective (Q1) (*n* = 7), did not locate the researcher culturally or theoretically (Q6) (*n* = 9), or address the researcher’s influence on the research and vice versa (Q 7) (*n* = 10). Despite these issues, the studies demonstrated strong methodological rigor overall. Appendix 2 provides the full list of questions, and Table 1 illustrates the critical appraisal results.

**Table 1:** Critical appraisal results

Citation	Q1	Q2	Q3	Q4	Q5	Q6	Q7	Q8	Q9	Q10
Boyd et al., 2011	U	Y	Y	Y	Y	N	N	Y	Y	y
Casey & Webb, 2021	Y	Y	Y	Y	Y	N	N	Y	Y	Y
Garcia et al., 2022	Y	Y	Y	Y	Y	Y	N	Y	Y	Y
Hennessy, 2015	Y	Y	Y	Y	Y	U	Y	Y	Y	Y
Hungerford et al., 2016	U	Y	Y	Y	Y	N	N	Y	Y	Y
Marina & Panoraia, 2024	Y	Y	Y	Y	Y	Y	Y	Y	Y	Y
Matscheck et al., 2020	U	Y	Y	Y	Y	N	N	Y	Y	Y
McCrae et al., 2008	Y	Y	Y	Y	Y	N	N	Y	U	Y
Osei, 2022	Y	Y	Y	Y	Y	Y	Y	Y	Y	Y
Shepherd & Meehan, 2013	U	Y	Y	Y	Y	N	N	Y	N	Y
Shepherd & Meehan, 2019	U	Y	Y	Y	Y	N	N	Y	Y	Y
Shepherd et al., 2014	U	Y	Y	Y	Y	N	N	Y	Y	Y
Taylor, 2015	Y	Y	Y	Y	Y	Y	Y	Y	Y	Y
Wilberforce et al., 2017	U	Y	Y	Y	Y	N	N	Y	Y	Y
%	50	100.0	100.0	100.0	100.0	28.57	28.57	100.0	85.71	100.0

Key: *Y* = Yes, *N* = No, *U* = Unclear

### Characteristics of Included Studies

Four articles (Hungerford et al., 2016; Shepherd & Meehan, 2013, 2019; Shepherd et al., 2014) were from Australia, two (Boyd et al., 2011; Garcia et al., 2022) from the United States of America, two (Hennessy, 2015; Taylor, 2015) from New Zealand, two from the United Kingdom (Marina & Panoraia, 2024; McCrae et al., 2008) and one each from Ireland (Casey & Webb, 2021), Canada (Osei, 2022), Sweden (Matscheck et al., 2020), and England (Wilberforce et al., 2017). Twelve articles were qualitative studies that used data collection methods appropriate to this form of inquiry, including interviews (Hennessy, 2015; Marina & Panoraia, 2024; Matscheck et al., 2020; Osei, 2022; Shepherd & Meehan, 2019; Taylor, 2015; Wilberforce et al., 2017), focus groups (Boyd et al., 2011; Garcia et al., 2022; Hungerford et al., 2016), interviews and focus groups (McCrae et al., 2008) and visual art-based narrative inquiry (Casey & Webb, 2021). The mixed methods studies (Shepherd & Meehan, 2013; Shepherd et al., 2014) used a combination of surveys and interviews or focus group discussions for data collection. Most studies used an inductive approach to thematic analysis of the findings. Table 2 illustrates the characteristics of the articles included.

**Table 2:** Characteristics of included studies

Study	Methods for data collection and analysis	Country	Phenomena of interest	Setting/context/culture	Participant characteristics and sample size	Description of main results
Boyd et al., 2011	Qualitative study Data collection method: Focus groups Data analysis: Content analysis, findings categorised into themes	United States of America	Staff practices, barriers, and facilitators in mental health referrals for women with depression within a community nonprofit agency serving low-income pregnant and postpartum women	Community-based private nonprofit organisation	The study participants were 16 female community health workers	1. Challenges in using the depression assessment scale and referral system 2. Client's lack of motivation 3. Language and cultural diversity 4. Developing a good relationship with clients and the staff at the mental health referral agencies
Casey &Webb, 2021	Qualitative study Data collection methods: Participative visual arts-based narrative inquiry following the guidelines for participatory visual research methodology (PVRM). Study participants constructed a mental image of their recovery and learning experience. They presented a visual art piece of their mental image to their classmates, describing the process involved in creating their art piece and what they were trying to communicate about their experiences. This was followed by classmates'responses to each presented art piece, commenting on how it resonated with them and their perspectives on the presented experiences Data analysis followed PVRM approaches, including individual and collective interpretations of the art piece and its meaning to them. This was followed by a secondary content and thematic analysis, including the art piece and the participants'interpretations	Ireland	Experiences and perceptions of mental health support workers regarding their mental health recovery work	The study was conducted as part of the training evaluation for a mental health recovery training program collaboratively developed by mental health practitioners, service users, and educationalists in Dublin, Ireland. The three participants whose arts narratives are presented in this article chose to concentrate on their perspectives and experiences of recovery in the context of their work roles as mental health support workers rather than on their learning journey in the program	The study participants were part of 14 female students undertaking mental health recovery training. The 14 students consented to participate in the initial arts-based narrative inquiry regarding their perceptions and experiences of learning and recovery. Two of the three participants had reported prior self-experience of mental health concerns. Total participants: Three support workers, two non-peer and one peer support worker	1. Recovery support 2. Belief in own knowledge and capacity to support those in mental distress 3. Occupational culture that hinders collaborative practice 4. Opposing views/practices of mental health recovery and helping relationships 5. Role confusion
Garcia et al., 2022	Qualitative- participatory action study Data collection methods: Focus groups	United States of America	Role of community health workers in meeting the mental health needs of Latino communities in Oregon, how they respond to challenging situations, and the training and support they require to be effective in their roles	Oregon- Community health workers from four geographical communities where Latinos live	The study participants were 92 Latino-certified community health workers working with Latino communities, 18 to 65 years old, and affiliated with a community health centre, hospital, or community organisation	1. lack of support from colleagues and administration 2. Cultural insensitivity among clinicians 3. Community health worker's safety
Hennessy, 2015	Qualitative study; an appreciative inquiry Data collection methods: Interviews which were recorded and transcribed Data analysis methods: Appreciative interpretation	New Zealand	To examine mental health support workers'contribution to the New Zealand mental health services	Auckland, Wellington, Hamilton and the South Island of New Zealand	The study participants were 34, including 20 non-peer mental health support workers, six peer support workers, two consumers, two mental health managers, one mental health educator, one family advisor and two other health professionals	1. Connection and relationship between mental health support workers and consumers 2. Role Clarity 3. Contribution of mental health support workers to mental health services 4. Support and supervision 5. Regulation and legislation of mental health support workers 6. Feeling valued 7. Health professional's awareness of mental health support workers'role 8. Collaborating with colleagues 9. Professionalisation of mental health support worker role 10. Career framework and progression 11. A consistent professional title 12. Adequate remuneration for mental health support workers 13. Conducive working environment
Hungerford et al., 2016	Qualitative study Data collection methods: Focus group discussions Data analysis: Data was coded and analysed inductively to identify emerging themes	Australia	To explore the benefits and challenges experienced by the community workers working with clinicians	The study was conducted in a major urban centre in southeastern Australia. Participants were employees of non-profit organisations that delivered community-based psychosocial services to people with serious mental illness	The study participants were employees of non-profit organisations that delivered community-based psychosocial services to people with serious mental illness. There were 15 community workers in total	1. A common understanding of consumers'recovery goals 2. Better collaboration between the community workers and clinicians improved consumer outcomes 3. Different understanding of the recovery concept 4. Different approaches to supporting consumer recovery 5. Relationship between the clinicians and the community workers 6. Misunderstanding of community workers'role by the clinicians
Marina & Panoraia, 2024	Qualitative study Data collection methods– Semi-structured interviews. Interviews were audio-recorded and transcribed verbatim Data analysis: Thematic analysis	United Kingdom	To explore the experiences of mental health support workers in independent/supported living facilities to improve their training, support and overall work experience	The study was conducted in the United Kingdom, and it involved mental health support workers working for private organizations	The study participants were 11 MHSWs, including four males and seven females	1. Utilization of a person-centred approach 2. Dealing with verbal abuse from service users 3. Role uncertainty and practical solutions 4. Promoting service users'independence 5. Availability of support from the employer
Matscheck et al., 2020	Qualitative study Data collection methods—interviews. Interviews were audio-recorded and transcribed in full Data analysis: Thematic analysis	Sweden	To identify the elements of user involvement from users’ and support workers’ descriptions of helpful support in daily living	Support services for people with severe mental illness in community care. The study was conducted in three municipalities	The study participants were 31, including 13 support workers (nine females and four males) and 18 consumers	1. Constant dialogue between the support workers and service users 2. Framing the flexibility of support worker roles 3. Importance of ‘small things’
McCrae et al., 2008	A mixed method study Data collection methods: Individual interviews, focus groups and surveys Data analysis: N6 qualitative data analysis software was used. The analysis included three processes: data reduction, data display, and conclusion drawing and verification. Qualitative findings were presented thematically	United Kingdom	To evaluate the introduction of support workers in community mental health teams for older adults	The study was conducted within a community mental health team in Lambeth, south London, following the introduction of support workers	The study participants were 28, including four support workers, 16 nurses, three psychiatrists, two psychologists, and three occupational therapists. All four support workers were interviewed after being in their role for six months and participated in a focus group after being in the role for one year; two nurses were included in the focus group. At 18 months of the role introduction, 23 staff were interviewed, including 16 nurses, three psychiatrists, two psychologists, and one occupational therapist	1. Lack of support workers'role clarity 2. Work assignment based on the professional practitioner's understanding of the support worker role 3. Valuable contribution to the team 4. Personal development opportunities
Osei, 2022	Qualitative study; phenomenological approach Data collection methods: Interviews Data analysis: Thematic analysis	Canada	The lived experiences of support workers providing support to people with schizophrenia and other mental health conditions in Winnipeg	Winnipeg, Manitoba	The study participants were mental health support workers hired by agencies to provide support services to people with schizophrenia in Winnipeg. They had a minimum of two years of experience. Total participants: Six mental health support workers, three males and three females	1. Barriers and facilitators related to support plans and their impact 2. Staff shortage and safety 3. Consumer's freedom of choice in decision-making
Shepherd & Meehan, 2013	Mixed methods research Data collection methods included questionnaires (paper or online format) and interviews. Interviews were conducted individually or in groups of up to four people Data analysis: The authors did not explicitly state their data analysis method; however, the quantitative results are presented in percentages, while the qualitative results appear to have been presented in themes	Australia	To establish the optimum level and content of training required by support workers to carry out their role effectively	The study was done in Queensland, Australia. The research was part of an evaluation of Project 300 (a supported housing program for people with serious mental illness)	Support workers working in non-government organisations funded to provide in-home support to Project 300 clients. The participants were required to have provided services to a Project 300 client for at least six months to be eligible to participate in the study. Total participants: 112 104 support workers completed questionnaires, and 18 (13 females and five males) participated in interviews. Eight managers were also interviewed	1. Need for training about the symptoms of mental illness, medications, and the recovery concept 2. Difficulty Motivating clients 3. Dealing with challenging behaviour
Shepherd & Meehan, 2019	Qualitative study Data collection methods: Interviews which were audio recorded and transcribed verbatim Data analysis: Theoretical analysis using NVivo 8; Iterative approach to data analysis, including first and second cycle methods. The first cycle used descriptive codes, and the second used theoretical codes	Australia	Division of labour between support workers employed by a non-government organisation and government health workers working within a cross-agency supported housing program for people with severe mental illness and at risk of homelessness and self-neglect	Queensland, Australia. Housing and support program (HASP)- a program developed to support people with mental illness who were at risk of homelessness and self-neglect. Care of clients was provided by staff employed by the government and staff from a non-government organisation	Study participants were support workers providing non-clinical support services to people in the HASP program, managers from non-government organisations, and case managers from government-funded mental health services. The total number of participants was 77, including 27 support workers, ten managers, and 40 case managers	1. Role boundaries: clinical role of case managers versus the non-clinical role of support workers 2. Maintenance of role boundaries; negotiating work roles 3. Tension between the support workers and the case managers due to poor communication, perceived lack of clinical intervention by case managers, role ambiguity, and support workers feeling that government health workers devalued their input and feedback
Shepherd et al., 2014	Mixed method study Data collection methods: Survey and semi-structured interviews Data analysis: Themes were identified via an inductive approach using NVivo 8	Australia	Challenges faced by in-home psychiatric support workers in implementing recovery in their work with clients with severe psychiatric disabilities	The study was in Queensland, Australia. The study was part of an evaluation of Project 300 (P300)—supported living in the community and Housing and Support Programme (HASP). The government funded non-government organisations to provide non-clinical support to clients in both programs	Participants were employees of non-government organisations contracted by the government to provide non-clinical support to clients. There were 37 total participants, 27 Support workers, and 10 Managers	1. Encouraging citizenship and establishing a working relationship; challenge finding a balance between encouraging client autonomy and the need for care to facilitate citizenship 2. Difficulty building a working relationship while working mainly in a client’s home, especially in establishing boundaries and worker safety
Taylor, 2015	Qualitative study: Feminist approach Data collection methods: Semi-structured interviews Data analysis method: Thematic analysis	New Zealand	To explore the experiences of mental health support workers from a feminist perspective	Regions across New Zealand	Study participants were community mental health support workers who worked or had previously worked in a non-government mental health service in New Zealand. There were 14 total participants, seven males and seven females	1. Low wages and lack of structured wage increase 2. Meaningful work satisfaction derived from working with clients 3. Blurred boundaries between formal and informal care work 4. Verbal abuse 5. Relationships with colleagues 6. Difficulties with the mental health system 7. High staff turnover 8. Supervision and support, training, and autonomy 9. Different work approaches 10. Staff safety 11. Hierarchy within the mental health organisations 12. Funding of non-governmental organisations
Wilberforce et al., 2017	Qualitative study Data collection methods: Semi-structured face-to-face interviews Data analysis: Thematic framework analysis	England	The functions of support workers in community mental health teams for older adults regarding roles, boundaries, supervision, and training	England	Participants were recruited from nine community mental health teams for older people geographically spread across England. Participants had a minimum of two years of experience in mental health. The total participants were 42, including five support workers, ten team managers, eight consultant psychiatrists, eight mental health nurses, five occupational therapists, two clinical psychologists and two social workers	1. Support workers undertook many roles and had significant autonomy over their duties 2. Negotiated boundaries that were occasionally breached 3. Support workers supported through open communication with the broader team 4. Limited training courses for support workers 5. Low pay for support workers

### Review Findings and Meta-aggregation

From the 14 articles included in this review, 116 findings illustrating the barriers and facilitators that MHSWs experience in their roles were extracted. The findings were grouped into 20 categories and resulted in four synthesized findings: 1) supportive work environment, 2) service user care barriers, 3) operational and support barriers, and 4) professional and role-related barriers.

### Synthesized Finding 1: Supportive Work Environment

**Fig. 2:**
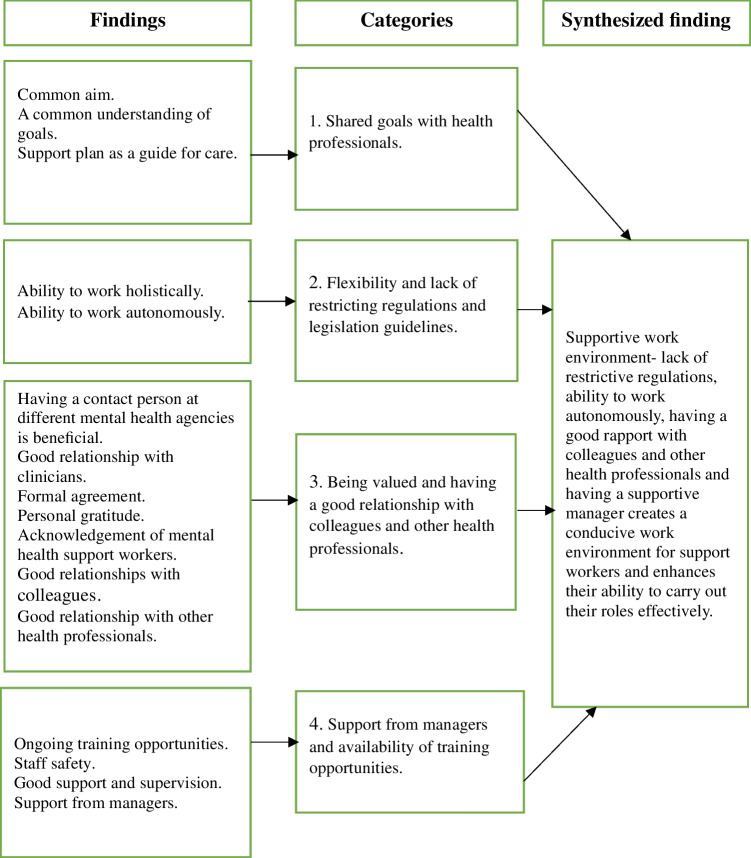
Supportive work environment

The work environment was viewed as supportive if it allowed support workers to work autonomously with limited restrictive regulations while encouraging positive relationships with colleagues and other healthcare professionals to facilitate effective collaboration. Additionally, a supportive manager who provided guidance and helped create a conducive working environment was perceived to be enabling MHSWs to carry out their roles effectively. This finding was generated from four categories (Fig. 2) and was supported by data from six studies (Boyd et al., 2011; Hennessy, 2015; Hungerford et al., 2016; Marina & Panoraia, 2024; Osei, 2022; Taylor, 2015).

#### Category 1: Shared Goals with health Professionals

MHSWs described shared goals with health professionals as important in promoting consistent and holistic care. They perceived that regular meetings and joint support planning enhanced collaboration with clinical staff (Hungerford et al., 2016). MHSWS also viewed recovery plans as useful tools that helped them better understand service users’ needs while providing them with access to relevant information (Hungerford et al., 2016; Osei, 2022).

#### Category 2: Flexibility and Lack of Restrictive Regulations and Legislation Guidelines

Although MHSWs reported they were working under the supervision of a case manager, they felt their roles were more flexible than those of other health professionals, whose practices MHSWs believed were constrained by inflexible professional regulations and legislation (Hennessy, 2015). This perceived flexibility was seen as enabling them to adapt their roles to the unique and evolving needs of each service user.

#### Category 3: Being Valued and Having Good Relationships with Colleagues and Other Health Professionals

Building a good rapport with other health professionals and being acknowledged for the skills and experience they brought to the team gave MHSWs a sense of belonging and recognition within multidisciplinary teams (Boyd et al., 2011; Hennessy, 2015; Hungerford et al., 2016; Taylor, 2015). They described how open communication and respectful relationships with clinicians fostered mutual learning and improved collaboration (Boyd et al., 2011; Hungerford et al., 2016). In some cases, MHSWs believed that formal agreements between their employing organizations and clinical mental health services improved collaboration, which they perceived as contributing to better recovery outcomes for the service users (Hungerford et al., 2016).

#### Category 4: Support from Managers and Availability of Training Opportunities

MHSWs valued regular supervision and guidance from managers, which they perceived as essential for maintaining a safe and supportive work environment (Taylor, 2015). They felt reassured if they had a manager they could turn to for assistance when facing challenges (Marina & Panoraia, 2024). Additionally, access to training opportunities was viewed as important for developing the knowledge and skills required to meet their role expectations and provide effective support (Taylor, 2015).

### Synthesized Finding 2: Service User Care Barriers

**Fig. 3:**
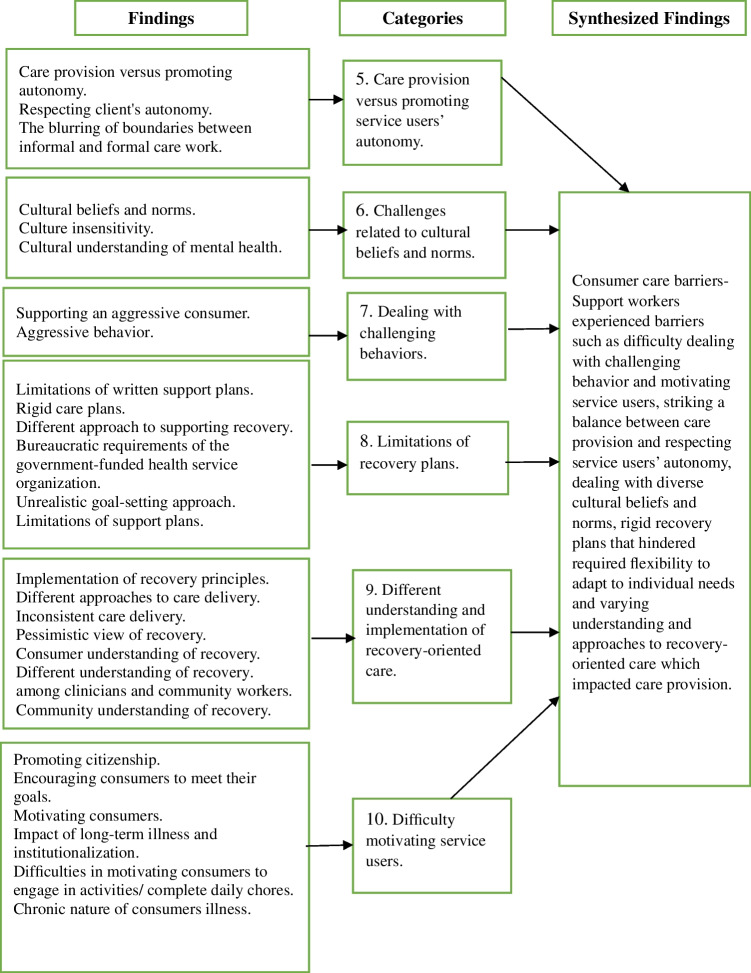
Service user care barriers

MHSWs described experiencing a range of barriers when providing support to service users in community settings (Fig. 3). The perceived barriers were drawn from ten studies (Boyd et al., 2011; Casey & Webb, 2021; Garcia et al., 2022; Hennessy, 2015; Hungerford et al., 2016; Matscheck et al., 2020; Osei, 2022; Shepherd & Meehan, 2013; Shepherd et al., 2014; Taylor, 2015) and included challenges such as managing challenging behavior, motivating service users, having a different understanding of recovery, and supporting autonomy while ensuring safety. These perceived challenges reflect the complexity of supporting individuals with diverse needs and fluctuating mental health in community settings where MHSWs often worked alone with limited formal structures. The barriers are grouped into six categories described below:

#### Category 5: Care Provision Versus Promoting Service Users’ Autonomy

MHSWs reported difficulties in maintaining a balance between supporting service users' independence and ensuring that tasks were completed when service users were reluctant to complete them or participate in community activities (Shepherd et al., 2014). While MHSWs understood their role as empowering service users, they also described situations where they felt obligated to complete tasks themselves to maintain a safe working environment. Additionally, when working with service users who experienced drug and alcohol issues, MHSWs expressed discomfort in reconciling service users’ autonomy with their own beliefs about health and wellbeing (Shepherd et al., 2014).

#### Category 6: Challenges Related to Cultural Beliefs and Norms

MHSWs reported experiencing challenges when working with some service users from culturally and linguistically diverse (CALD) backgrounds, mainly when they had different understandings of mental illness compared to how it is described and understood in Western medicine (Hennessy, 2015). They described instances where service users from CALD communities were hesitant to discuss mental health concerns or allow MHSWs into their homes, which was perceived as a barrier to establishing trust and providing support (Boyd et al., 2011; Hennessy, 2015).

#### Category 7: Dealing with Challenging Behaviors

MHSWs reported difficulties in managing behaviors such as non-concordance with medication, expressions of anger, and refusal to engage in activities (Shepherd & Meehan, 2013; Shepherd et al., 2014). These behaviors were described as particularly difficult to manage in isolated work settings, such as the service user’s home, where MHSWs often worked alone and felt vulnerable (Taylor, 2015).

#### Category 8: Limitations of Recovery Plans

While MHSWs generally saw recovery plans as helpful in ensuring that they provided structured and consistent support, some felt the plans lacked the flexibility to respond to the dynamic nature of service users’ lives (Hungerford et al., 2016; Matscheck et al., 2020; Osei, 2022; Taylor, 2015). This perceived inflexibility was described as a barrier to supporting recovery in a person-centred and responsive way.

#### Category 9: Different Understanding and Implementation of Recovery-oriented Care

MHSWs reported that recovery-oriented care was understood and applied differently across individuals, teams and organisations. While most organisations promoted recovery principles in their values, MHSWs perceived inconsistencies in how these values were translated into everyday practice (Taylor, 2015). This inconsistency reportedly contributed to varied approaches to care delivery across workers and settings, which some MHSWs found challenging when trying to implement recovery-oriented interventions (Taylor, 2015).

MHSWs also reported differing interpretations of recovery among themselves, clinical staff, service users, and the broader community (Hungerford et al., 2016). They described feeling confident in their understanding of recovery-oriented practice but experienced tensions when working with clinicians who were perceived to adopt biomedical views of recovery (Casey & Webb, 2021). Additionally, they reported that it was difficult to explain the meaning of recovery to some service users, especially those from CALD backgrounds, whose cultural understanding of illness and healing did not always align with the mainstream recovery frameworks (Hungerford et al., 2016).

#### Category 10: Difficulty Motivating Service Users

MHSWs encouraged service users to set goals to inspire hope in their recovery journey, but it was challenging at times to encourage goal-oriented behavior due to the fluctuating nature of the service users'mental health issues (Shepherd & Meehan, 2013; Shepherd et al., 2014; Taylor, 2015). They also perceived that applying recovery-oriented principles was particularly challenging when supporting individuals with long-term mental health conditions and extensive histories of institutional living. They described that some service users, while expressing hopes or dreams, appeared hesitant to take action toward change, often due to past disappointments or a preference for more familiar routines. In these situations, MHSWs viewed recovery as a process where progress could be reflected in small but meaningful changes, such as becoming more flexible and spontaneous in the things that the service users did, rather than significant behavioral or lifestyle changes (Shepherd & Meehan, 2013).

### Synthesized Finding 3: Operational and Support Barriers

**Fig. 4:**
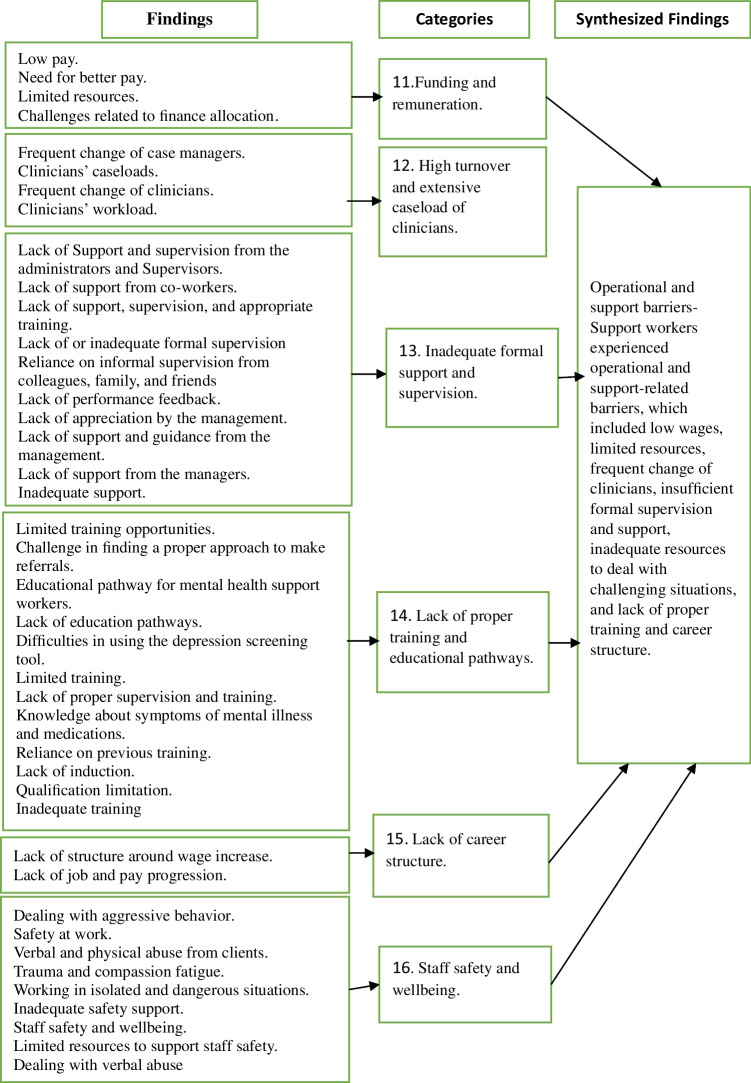
Operational and support barriers

This finding was synthesized from data derived from 11 studies (Boyd et al., 2011; Garcia et al., 2022; Hennessy, 2015; Hungerford et al., 2016; Marina & Panoraia, 2024; McCrae et al., 2008; Osei, 2022; Shepherd & Meehan, 2013, 2019; Taylor, 2015; Wilberforce et al., 2017). The operational and support barriers (Fig. 4) included concerns about remuneration, inadequate training and supervision, limited resources, and workplace safety. The issues are grouped into six categories described below.

#### Category 11: Funding and Remuneration

MHSWs perceived their wages as low, compared to workers in other professions, and did not reflect their job responsibilities or the risks and challenges they face (Hennessy, 2015; Taylor, 2015). They expressed the need to be recognized and compensated appropriately. Additionally, limited funding was perceived to affect service quality, with MHSWs reporting that insufficient resources made it difficult to deliver their services effectively (Taylor, 2015).

#### Category 12: High Turnover and Extensive Caseloads of Clinicians

MHSWs reported difficulties in collaborating with clinical staff, particularly due to the frequent turnover of case managers (Hungerford et al., 2016; Shepherd & Meehan, 2019). Some MHSWs reported not being informed when a service user’s case manager changed, which they perceived as disrupting the continuity of care. MHSWs attributed these issues to high clinician caseloads, which they perceived as limiting communication and coordination between the services (Hennessy, 2015; Hungerford et al., 2016).

#### Category 13: Inadequate Formal Support and Supervision

MHSWs reported not receiving regular or structured supervision to help them manage the emotional and psychological demands of their work (Garcia et al., 2022; Taylor, 2015). In the absence of formal support structures, they reported relying on informal support networks such as colleagues, friends, or family members to discuss work-related challenges (Taylor, 2015).

#### Category 14: Lack of Proper Training and Educational Pathways

MHSWs expressed concerns about insufficient training and preparation for the wide range of tasks involved in their roles. The studies did not identify the mental health training received but MHSWs articulated a lack of, or limited availability of specialized training tailored to their role which made them feel underprepared to recognize or respond to certain symptoms, medication issues, or physical health concerns among service users (Boyd et al., 2011; Shepherd & Meehan, 2013; Taylor, 2015). Additionally, training provided by the employer was perceived as too generic or lacking a practical application to their role, which some felt contributed to reduced confidence in their ability to manage complex situations (Hennessy, 2015; Marina & Panoraia, 2024; McCrae et al., 2008; Taylor, 2015; Wilberforce et al., 2017).

#### Category 15: Lack of Career Structure

An absence of clear career progression or structured pathways for advancement was reported by MHSWs (Taylor, 2015). The lack of progression was perceived as contributing to feelings of being undervalued and, for some, led to job dissatisfaction, which increased their intention to leave their role.

#### Category 16: Staff Safety and Well-being

MHSWs reported feeling unsafe at times, recounting experiences of verbal abuse, threatening behavior and occasionally, physical aggression from service users (Garcia et al., 2012; Osei, 2022; Taylor, 2015). While some MHSWs reported managing these challenges by rationalizing the abuse based on the service user’s presentation and remaining professional without taking things personally (Marina & Panoraia, 2024), others reported that the emotional toll of their role impacted their mental health and well-being, and they felt that they lacked adequate organizational support to manage these effects (Garcia et al., 2022; Taylor, 2015).

### Synthesized finding 4: Professional and Role-related Barriers

**Fig. 5:**
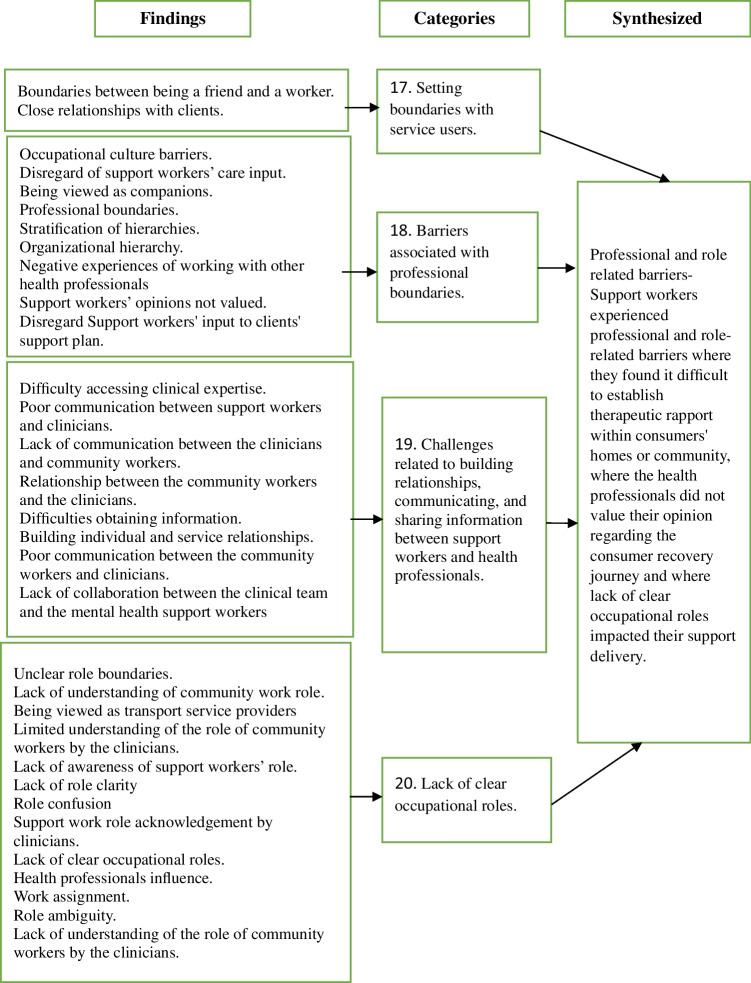
Professional and role related barriers

This synthesized finding reflects MHSWs perceptions of challenges related to establishing professional boundaries with service users and healthcare professionals, as well as ambiguity surrounding their roles and responsibilities within the mental health system. These challenges were drawn from nine studies (Casey & Webb, 2021; Hennessy, 2015; Hungerford et al., 2016; Marina & Panoraia, 2024; McCrae et al., 2008; Osei, 2022; Shepherd & Meehan, 2019; Shepherd et al., 2014; Taylor, 2015) and are presented in four categories (Fig. 5).

#### Category 17: Setting Boundaries with Service Users

There was a recognition that developing a therapeutic relationship was an important part of being able to work in collaboration with service users. MHSWs reported that establishing therapeutic relationships in community settings, particularly within service users’ homes, involved navigating relational boundaries that were often less defined than in clinical environments (Shepherd et al., 2014). They described how some service users viewed them as friends or companions, mainly due to the informal nature of their interactions and being consistently present in their day-to-day lives (Shepherd et al., 2014). While some MHSWs saw this closeness as helpful in building trust, others were concerned that it might unintentionally hinder service users’ motivation to engage socially beyond the support relationship, hindering the broader recovery goal of social inclusion. Boundaries in this context were about negotiating a relational connection that was therapeutic and supportive, without reinforcing dependency.

#### Category 18: Barriers Associated with Professional Boundaries

MHSWs perceived that other healthcare professionals often undervalued their contributions to service users'recovery-oriented care (Casey & Webb, 2021; Hennessy, 2015; Hungerford et al., 2016; Osei, 2022; Taylor, 2015). While they were the ones involved in the day-to-day support of service users, they felt that they could make valuable contributions related to care planning or the person’s recovery journey, but were often unappreciated or ignored by clinical staff. MHSWs also felt that clinical staff viewed them as companions to service users, rather than as members of the care team. This perceived role ambiguity created a sense of marginalization and reinforced hierarchical divisions within mental health teams (Casey & Webb, 2021) which not only diminished MHSWs morale but was also seen as a barrier to information sharing and service coordination.

#### Category 19: Challenges Related to Building Relationships, Communicating, and Sharing Information Between Support Workers and Health Professionals

MHSWs reported difficulties in building collaborative relationships and sharing information with clinical staff (Hennessy, 2015; Hungerford et al., 2016; Shepherd & Meehan, 2019). Even though they were required to work alongside healthcare professionals, MHSWs felt that communication and partnership were often lacking. They expressed a desire for stronger, more inclusive collaboration that would support more coordinated and holistic care for the service users (Hennessy, 2015; Hungerford et al., 2016).

#### Category 20: Lack of Clear Occupational Role

The challenges in developing relationships, communicating, and sharing information with the healthcare team may be connected to a lack of understanding of the role of the MHSW among organizations, clinical teams and MHSWs themselves. In five papers, MHSWs reported a lack of clarity around their roles and responsibilities within the mental health system (Casey & Webb, 2021; Hungerford et al., 2016; Marina & Panoraia, 2024; McCrae et al., 2008; Shepherd & Meehan, 2019). This ambiguity was reported to contribute to misunderstanding and tension between themselves and the health professionals. MHSWs articulated that clearly defined roles would enhance their confidence, reduce role conflict, help others to better understand their role and improve their ability to contribute effectively within multidisciplinary teams (Hungerford et al., 2016; McCrae et al., 2008).

## Discussion

This systematic review synthesized existing qualitative evidence on the experiences of MHSWs to identify the barriers and facilitators they face in their roles. While some facilitators, such as flexible work environments and supportive professional relationships, were evident, the majority of the findings were barriers that hinder MHSWs ability to carry out their roles effectively and limit implementation of recovery-oriented care in practice. To position the discussion of this review within the contemporary recovery-oriented care, it is important to distinguish between clinical recovery, which focuses on measurable disease outcomes, such as the presence or absence of symptoms and medication compliance, often defined from a professional or service-oriented perspective (Davidson et al., 2005) and personal recovery, which is consumer-driven and prioritizes well-being, autonomy, identity, hope, and community connection, even in the ongoing presence of mental health symptoms (Slade, 2009). Recovery is not simply a set of practices, but a personal, non-linear process shaped by the individual’s own values and goals.

While MHSWs are well-positioned to support personal recovery through relational and practical support, their roles remain inconsistently defined, poorly integrated, and insufficiently supported within mainstream mental health services. Additionally, the interpretation and application of recovery-oriented principles vary across disciplines and settings, including within the research literature itself. Notably, some of the studies included in this review employed language that was not fully aligned with recovery-oriented practice, reflecting broader systemic inconsistencies. These findings reflect the complexity of embedding non-clinical, recovery-supportive roles within a mental health system that is still mainly shaped by hierarchical biomedical and risk-focused paradigms. The discussion of the review findings is organized around five interrelated themes: (1) work environment, (2) balancing care provision and service user autonomy, (3) remuneration, professional development and supervision challenges, (4) workplace safety and aggression management and (5) role clarity and relationship boundaries. Together, these themes offer an insight into the factors that influence the effectiveness of MHSWs roles in delivering recovery-oriented care.

### Work Environment

A supportive work environment was identified in this review as a key facilitator that enabled MHSWs to carry out their roles effectively. Key features of such an environment included autonomy and role flexibility, working collaboratively towards shared goals with health professionals, good relationships with colleagues and other health professionals, and a supportive manager. These findings are consistent with a recent umbrella review on the effectiveness, implementation and experiences of peer support approaches for mental health (Cooper et al., 2024) which identified supervision, strong leadership, a supportive and trusting workplace culture and effective collaboration with clinical teams as key factors to successful implementation of support work roles in mental healthcare. Peer support workers are non-clinical care providers like MHSWs, but they have personally experienced mental health issues and, therefore, use their own experiences to support other service users who are struggling with mental health conditions (Clossey et al., 2018). While the roles of these two groups of workers are usually presented as distinct from each other, both roles are aimed at supporting mental health service users in their recovery process, and they appear to benefit from similar organizational conditions. In both roles, clarity around contributions to recovery-oriented care is crucial for effective collaboration with clinical teams.

A supportive work environment for MHSWs was also associated with increased job satisfaction (Hennessy, 2015; Taylor, 2015), provision of holistic care (Hennessy, 2015; Hungerford et al., 2016), development of positive relationships with colleagues and health professionals (Hennessy, 2015), and enhanced teamwork and collaborative care (Hennessy, 2015). Similar benefits have been reported by nurses and other health professionals in various healthcare contexts (Aiken et al., 2012; Berta et al., 2018; Bronkhorst et al., 2015; López Gómez et al., 2019; Wood et al., 2011). While job roles and responsibilities may differ across professions and settings, there appears to be a shared understanding among the healthcare workforce of the importance of a positive and supportive environment. For MHSWs, fostering such conditions not only promotes job satisfaction but also strengthens collaboration with clinical teams, ultimately leading to more effective and responsive, recovery-oriented care for service users.

### Balancing Care Provision and Service User Autonomy

A key challenge to MHSWs role was balancing care provision and promoting autonomy, particularly when service users are reluctant to complete tasks or participate in community activities. This was compounded by the difficulty in motivating service users and dealing with challenging behaviours such as anger and refusal to engage in activities. These findings illustrate that, although recovery-oriented practice calls for a shift toward service user autonomy and collaborative decision-making (Davidson et al., 2007), these ideals are not always easily operationalised. In one study, Shepherd et al. (2014) described instances where MHSWs performed household duties for service users to ensure a safe work environment, even though they ideally wanted to promote the service user’s independence. Additionally, some MHSWs found it challenging to respect service users’ choices when those choices contradicted their own beliefs about health or risk.

The tension between the care provision and promoting service user autonomy has been highlighted in other studies (Hawkins et al., 2011; Hertzberg et al., 2023; Sandhu et al., 2017) and often intersects with the philosophical discussions on risk, agency and person-centred care. While traditional care models may focus on risk management and elimination of mental health symptoms, recovery-oriented care requires a shift towards empowerment, which can be complex given the often-fluctuating nature of serious mental health conditions (Davidson et al., 2007; Slade, 2009). Strategies that can be used to manage this tension and motivate service users in their recovery journey, such as involving service users in planning their services (Krotofil et al., 2018), persuasive communication and building trust with service users (Hertzberg et al., 2023) and creating a relaxed, friendly, and non-judgmental environment (Chen & Oh, 2019) have also been suggested in the literature. Although their effectiveness has not been measured, qualitative evidence indicates that these strategies can enhance service user engagement, promote independence and improve overall care outcomes (Chen & Oh, 2019; Hertzberg et al., 2023; Krotofil et al., 2018).

The conflict between the duty of care and enhancing service users'autonomy was further compounded by cultural diversity, as our review identified a lack of confidence among MHSWs in delivering care to service users from CALD backgrounds. Differences in how mental illness is understood and discussed across cultures were identified as barriers to building trust and establishing effective therapeutic relationships (Boyd et al., 2011; Hennessy, 2015). Literature has highlighted the need to provide cultural competence training to MHSWs to improve the service users'engagement and experience (Hoeft et al., 2018). Creating MHSWs cultural awareness through tailored training programs can provide them with the knowledge and skills necessary for them to deliver culturally responsive care to people from diverse cultural and linguistic backgrounds (Garcia et al., 2022).

While recovery-oriented care serves as the foundation for promoting autonomy in mental health care, it was identified to be understood and implemented variably across different roles and organizations. While most MHSWs described themselves as working from a recovery-oriented perspective, they perceived inconsistencies in how their colleagues and organizations interpreted and applied recovery principles. Slade et al. (2008) highlight that while recovery is widely referenced in policy, it is often operationalized differently in practice. This variability can create inconsistencies in how recovery is experienced by service users and supported by healthcare workers, including MHSWs (Saari et al., 2017). To embed recovery in practice, it is important to cultivate a culture that supports recovery and is dedicated to integrating recovery values into all organizational processes, with Slade et al. (2008) calling for a fundamental shift in the values of mental health services to achieve this goal. To support MHSWs perform their roles effectively, there is a need for a whole mental health system approach to clarify roles, embed recovery values in supervision and leadership structures, and promote interdisciplinary collaboration and coordination of care. These will not only support MHSWs in navigating the complexities of autonomy and care provision but also ensure that recovery-oriented principles are effectively integrated into mental health services.

### Remuneration, Professional Development, and Supervision Challenges

Our review identified a disparity in the actual compensation of support workers and the perceived value of their work. The findings reveal that MHSWs often feel their pay does not reflect their broad and complex job responsibilities. This sentiment is compounded by the lack of a clear career and pay progression structure, which results in pay variations across organisations. Cooper et al. (2024) identified similar perceptions among peer support workers. Additionally, low pay and limited career progression opportunities have been identified as significant factors in healthcare workforce turnover and intention to leave (Bimpong et al., 2020).

The review findings also highlight that MHSWs lacked access to formal supervision and relied on informal support from peers, family, or friends to manage work-related stress (Garcia et al., 2022; Taylor, 2015). The lack of structured guidance was reported to affect confidence, well-being, and the capacity to manage complex or emotionally demanding situations (Marina & Panoraia, 2024). These experiences point to the need for consistent access to reflective supervision, particularly given the relational and emotionally intensive nature of support work.

In terms of training, MHSWs reported feeling underprepared to manage some aspects of their roles, including recognizing and responding to mental health symptoms, medication-related issues, and physical health needs (Boyd et al., 2011; Shepherd & Meehan, 2013). While MHSWs’ qualifications and training vary widely across settings (Rifkin, 2017; WHO, 2022), many support workers do not have formal mental health qualifications. Mostly, employers tend to prioritize previous experience in care roles or provide brief in-house training (Marina & Panoraia, 2024; Tudor et al., 2018). While some staff may hold qualifications such as a Certificate IV in mental health in Australia, there is no consistent educational pathway or accreditation for this workforce (Manthorpe et al., 2010). The lack of standardization contributes to variability in competency and role expectations, as well as tensions with other members of multidisciplinary teams (Saari et al., 2017). Despite the limitations, MHSWs play an important role in promoting service users’ self-efficacy, empowerment, and recovery (Wilberforce et al., 2017), which can complement the work of staff from health and social care. As such, strengthening the role through the standardization of core competencies, creation of accredited training pathways, and formalized supervision structures could help enhance the sustainability of the role and improve service user outcomes. The importance of providing adequate training, support and supervision for support workers is highlighted in the literature as these are key to building confidence and increasing intention to stay in the role (Cooper et al., 2024; Saari et al., 2017). Additionally, training is associated with high intrinsic motivation and self-efficacy (Mirbahaeddin & Chreim, 2022).

### Workplace Safety and Aggression Management

Our review findings indicate that MHSWs encounter aggressive behaviour frequently. These findings are consistent with previous research that highlighted high levels of exposure to verbal and physical aggression among community-based workers (Kadri et al., 2018; Womack et al., 2020). Although mental illness might contribute to the risk of aggression for some service users (Caruso et al., 2021), other environmental and interpersonal factors also play a role in mitigating or escalating these incidents. The need for personal space and the interpersonal and communication skills of staff may play a role in reducing the risk of experiencing or de-escalating aggression (Cutcliffe & Riahi, 2018).

Literature also suggests that targeted communication and management of aggression training can help MHSWs feel more confident and better equipped to manage challenging behaviours (Baby et al., 2018; Richter et al., 2006). However, few of the studies in this review reported MHSWs having access to such training. Given the safety risks and emotional impact associated with these experiences, it is important that MHSWs receive not only practical training but also structured organizational support. Regular supervision and professional development initiatives could ensure that MHSWs are equipped with effective de-escalation techniques while also addressing the psychological impact of managing challenging behaviors (Garcia et al., 2022; Marina & Panoraia, 2024). Strengthening these supports would not only enhance the well-being of MHSWs but also improve service quality and safety for both staff and service users.

### Role Clarity and Relationship Boundaries

Role ambiguity was a recurring challenge identified across the studies reviewed. This review found that unclear occupational roles can restrict MHSWs interventions or result in the delegation of duties and responsibilities beyond their expertise. These findings are consistent with those of Saari et al. (2017), who observed that the absence of clear occupational roles and uncertainty surrounding the transfer of skills to personal support workers impacted the delegation process. Clear role clarification is associated with high levels of job satisfaction and lower rates of staff turnover (Hassan, 2013), while unclear job descriptions have been linked to uncertainty about role responsibilities, inadequate support and training and feeling undervalued (Cooper et al., 2024). To ensure that MHSWs perform their roles safely and effectively, it is essential that organisations provide formal role definitions, outline responsibilities and provide support that acknowledges the complexity of their role and integrates them as valued members of the multidisciplinary health and social care teams. Strengthening these foundations will not only improve workforce stability but also enhance the quality of care delivered to service users.

The blurring of boundaries also emerged in relation to therapeutic relationships. MHSWs reported challenges in establishing therapeutic relationships within the homes of service users who viewed them as friends. Research shows that people with mental illness are more likely to experience social isolation compared to the general population (Linz & Sturm, 2013). As such, the social bond between a support worker and a service user can be viewed as a form of friendship, which could fulfil the service user’s longing for meaningful connections. While close relationships could be helpful in building trust, MHSWs were concerned that being viewed as a friend may hinder service users from developing their own friendships (Shepherd et al., 2014). However, research suggests that professional support complements the ability to develop natural relationships (Tsai et al., 2012). To navigate this balance effectively, MHSWs require specific skills and structured guidance on using their rapport with service users to promote autonomy and social engagement. Providing training in boundary-setting and community integration strategies could equip MHSWs with techniques to encourage service users to form independent connections within their communities. Additionally, fostering social interactions and practical support structures can help people with serious mental illness rebuild relationships with family, friends, and broader social networks (Raphael-Greenfield & Gutman, 2015). Ensuring MHSWs have the skills to support service users in expanding their social circles without becoming their sole source of companionship is critical to enhancing recovery-oriented care.

### Limitations

While this review was conducted with significant methodological rigor, the findings have potential limitations. First, the review included primary research articles only; therefore, findings from other types of articles, such as reviews, editorials, and commentaries, were excluded. Second, the review included articles in English; thus, articles in other languages that may have contributed to an understanding from different cultural perspectives might have been excluded. Third, the perspectives and experiences of other mental health stakeholders, such as service users, carers, and other health professionals, are important in understanding the barriers to and facilitators of MHSWs roles; however, the phenomena of interest for this review were the experiences and perspectives of non-peer MHSWs.

### Implications for Policy and Practice

As highlighted throughout the discussion, this review identified several implications to support the effective implementation of MHSWS roles.

First, workforce development is a key area that requires attention. MHSWs require access to ongoing, structured training in mental health, cultural responsiveness, recovery principles, and de-escalation techniques. These should be paired with regular supervision that provides both professional guidance and emotional support, given the relational and often complex nature of support work.

Second, role clarity is essential for safe and effective practice. Formal definitions of the MHSW role, including core competencies, responsibilities and ethical boundaries, are needed to reduce ambiguity and ensure consistent practice across services. Formal recognition of MHSWs as a distinct non-clinical workforce that complements clinical care is key to improving collaboration and job satisfaction. Adequate remuneration and career progression structures are also necessary for workforce retention.

Third, workplace safety and boundary setting need to be strengthened through training and organizational support. MHSWs should be equipped to manage aggression and navigate close therapeutic relationships while maintaining appropriate boundaries that support service user autonomy and social connectedness.

Finally, MHSWs need to be integrated into multidisciplinary mental health teams through formal mechanisms for communication, shared care planning, and interprofessional education. Incorporating MHSWs into broader mental health workforce strategies will ensure that their contributions to recovery-oriented, person-centred care are fully realized across service settings.

### Implications for Research

This review identified the barriers to and facilitators of the MHSWs role from the perspective of MHSWs only. To provide a holistic understanding of the challenges and facilitators, along with their impact on the implementation of services delivered by MHSWs, future research should include the perspective of other key stakeholders, including service users, caregivers, and clinical professionals. This may identify other determinants of service delivery by MHSWs and service users’ outcomes.

Although the concept of recovery is embedded in mental health policy, evidence suggests that its practical application is inconsistent. To address this, future research can explore how factors such as organizational cultures, funding models such as the National Disability Insurance Scheme (NDIS) in Australia, professional boundaries and risk aversion shape how recovery is interpreted and operationalized in daily practice.

Equally important is research that investigates the relational dimensions of MHSW roles and the impact this has on service users experience. Building trust, navigating ambiguous boundaries, and fostering social inclusion without reinforcing dependency are core to supporting recovery, but there appears to be a lack of empirical evidence in this area compared to clinical interventions.

The review also highlighted several challenges in the collaboration between MHSWs and other healthcare professionals, including issues related to professional boundaries, communication, and role recognition, which can negatively impact service coordination and the continuity of recovery-oriented care. Future research could explore the dynamics of collaboration and care coordination across hospitals, primary care, and community mental health services, with a focus on integrating MHSWs into multidisciplinary teams. Investigating how collaborative processes shape recovery-oriented service delivery could provide insights into best practices. Furthermore, the outcomes could facilitate a more seamless continuation of recovery-oriented care for service users.

## Conclusion

The findings of this review provide insights into the factors that determine the effective delivery of MHSWs roles. While MHSWS are well-positioned to provide relational, community-based care that aligns with the principles of personal recovery, such as hope, autonomy, and social inclusion, the findings reveal a persistent mismatch between these aspirations and the realities of practice. These challenges not only affect the confidence and effectiveness of MHSWs but also limit the potential of recovery-oriented practice to be meaningfully embedded in routine care. The findings show that MHSWs currently face more barriers than facilitators, which need to be addressed to maximize the potential of this role in supporting service users.

Recovery is a complex framework that is operationalized through a values-based orientation that must be lived through relationships, flexibility, and co-production. Without clear structural investment, reflective practice, and interprofessional collaboration, there is a risk of MHSWs being marginalized within mental health care. Ultimately, the review findings highlight the need to move beyond rhetoric and foster environments where MHSWs can work safely, collaboratively, and with a shared purpose, thereby contributing fully to the implementation of person-centred, recovery-oriented care that enhances the service user experience.

## Supplementary Information

Below is the link to the electronic supplementary material.

Supplementary file1 (DOCX 443 KB)
