# Supplementary material for: Mental Health Support Workers Perspectives on Barriers to and Facilitators of the Effective Delivery of their Roles: A Systematic Review and Meta-aggregation
Source: Community Ment Health J. 2025 Jul 24;61(8):1548–73. doi: 10.1007/s10597-025-01490-9 (PMC12647239; doi:10.1007/s10597-025-01490-9)
Supplement: Supplementary file 1 — Supplementary file1 (DOCX 443 KB) [file 10597_2025_1490_MOESM1_ESM.docx]

# Appendices

## Appendix 1: Search strategy

|  |  | MEDLINE | CINAHL | PsycINFO | Web of Science  Search field: Abstract |
| --- | --- | --- | --- | --- | --- |
| 1 | "Support worker*" | 1,655 | 1,954 | 1,224 | 1,984 |
| 2 | "Peer support worker*" | 315 | 247 | 244 | 344 |
| 3 | "Support worker*" NOT "Peer support worker*" | 1,340 | 1,707 | 976 | 1,640 |
| 4 | (MH "Community Health Workers") | 7185 | 5,122 | 783 | 6,703 |
| 5 | "Health care assistant*" | 397 | 457 | 118 | 280 |
| 6 | "Health care aide*" | 80 | 74 | 43 | 67 |
| 7 | (MH "Home Health Aides") | 853 | 1,644 | 142 | 239 |
| 8 | "Lay health worker*" | 552 | 274 | 198 | 454 |
| 9 | "Community health aide*" | 81 | 21 | 20 | 32 |
| 10 | (MH "Psychiatric Aides") | 414 | 47 | 193 | 18 |
| 11 | "Mental Illness*" | 51,842 | 28,695 | 62,601 | 43,948 |
| 12 | (MH "Mental Disorders") | 186,501 | 72,670 | 152,257 | 53,634 |
| 13 | (MH "Mental Health") | 72,725 | 70,633 | 113,994 | 267,579 |
| 14 | "Severe mental illness*" | 6,894 | 3,701 | 7,229 | 6,104 |
| 15 | "Serious mental illness*" | 5,687 | 3,612 | 10,526 | 4,976 |
| 16 | "Enduring mental illness*" | 135 | 166 | 166 | 114 |
| 17 | "Psychiatric patient*" | 15,816 | 18,719 | 40,033 | 10,004 |
| 18 | “Psychiatric disability” | 453 | 274 | 788 | 379 |
| 19 | "Mental health rehabilitation" | 264 | 180 | 584 | 175 |
| 20 | (MH "Rehabilitation") | 18,882 | 20,317 | 25,622 | 220,814 |
| 21 | (MH "Psychiatric Rehabilitation") | 814 | 820 | 199 | 845 |
| 22 | (MH "Community Mental Health Services") | 19,364 | 11,714 | 16,156 | 1,111 |
| 23 | Recovery | 701,921 | 131,850 | 93,494 | 998,529 |
| 24 | (MH "Mental Health Services") | 41,352 | 40,387 | 52,122 | 27,873 |
| 25 | 3 OR 4 OR 5 OR 6 OR 7 OR 8 OR 9 OR 10 | 10,556 | 9.117 | 2,400 | 9,371 |
| 26 | 11 OR 12 OR 13 OR 14 OR 15 OR 16 OR 17 OR 18 | 284,274 | 161,795 | 297,288 | 338,993 |
| 27 | 19 OR 20 OR 21 OR 22 OR 23 OR 24 | 778,029 | 200,048 | 177,462 | 1,221,407 |
| 28 | 25 AND 26 AND 27 | 139 | 111 | 128 | 175 |
| 29 | ENGLISH ARTICLES | 136 | 109 | 127 | 175 |

| DATABASE | CONCEPT 1 | CONCEPT2 | CONCEPT3 | RESULT |
| --- | --- | --- | --- | --- |
| MEDLINE | ("Support worker*" NOT "Peer support worker*") OR (MH "Community Health Workers") OR "Health care assistant*" OR "Health care aide*" OR (MH "Home Health Aides") OR "Lay health worker*" OR "Community health aide*" OR (MH "Psychiatric Aides") | (MH "Mental Disorders") OR "Mental Illness*" OR (MH "Mental Health") OR "Severe mental illness*" OR "Serious mental illness*" OR "Enduring mental illness*" OR "Psychiatric patient*" OR "Psychiatric disability" | (MH "Community Mental Health Services") OR (MH "Mental Health Services") OR "Mental health rehabilitation" OR (MH "Psychiatric Rehabilitation") OR (MH "Rehabilitation") OR Recovery | 136 |
| CINAHL | ("Support worker*" NOT "Peer support worker*") OR MH "Community Health Workers" OR "Health care assistant*" OR "Health care aide*" OR (MH "Home Health Aides") OR "Lay health worker*" OR "Community health aide*" OR "Psychiatric Aide*" | "Mental Illness*" OR (MH "Mental Disorders") OR (MH "Mental Health") OR "Severe mental illness*" OR "Serious mental illness*" OR "Enduring mental illness*" OR "Psychiatric patient*" OR “Psychiatric disability” | (MH "Community Mental Health Services") OR (**MH** "Mental Health Services") OR "Mental health rehabilitation" OR "Psychiatric Rehabilitation" OR (MH "Rehabilitation") OR Recovery | 109 |
| PSYCINFO | ("Support worker*" NOT "Peer support worker*") OR (DE "Community Health Workers") OR "Health care assistant*" OR "Health care aide*" OR (DE "Home Health Aides") OR "Lay health worker*" OR "Community health aide*" OR (DE "Psychiatric Aides") | (DE "Mental Disorders") OR "Mental Illness*" OR (DE "Mental Health") OR "Severe mental illness*" OR "Serious mental illness*" OR "Enduring mental illness*" OR "Psychiatric patient*" OR "Psychiatric disability" | (DE "Community Mental Health Services") OR (DE "Mental Health Services") OR "Mental health rehabilitation" OR (DE "Psychiatric Rehabilitation") OR (DE "Rehabilitation") OR Recovery | 127 |
| WEB OF SCIENCE | AB=(("Support worker*" NOT "Peer support worker*") OR "Community Health Worker*" OR "Health care assistant*" OR "Health care aide*" OR "Home Health Aide*" OR "Lay health worker*" OR "Community health aide*" OR "Psychiatric Aide*") | AB=("Mental Disorder*" OR "Mental Illness*" OR "Mental Health" OR "Severe mental illness*" OR "Serious mental illness*" OR "Enduring mental illness*" OR "Psychiatric patient*" OR "Psychiatric disability" ) | AB=("Community Mental Health Service*" OR "Mental Health Service*" OR "Mental health rehabilitation" OR "Psychiatric Rehabilitation" OR Rehabilitation OR Recovery) | 175 |

## Appendix 2: Critical Appraisal Checklist for Qualitative Research


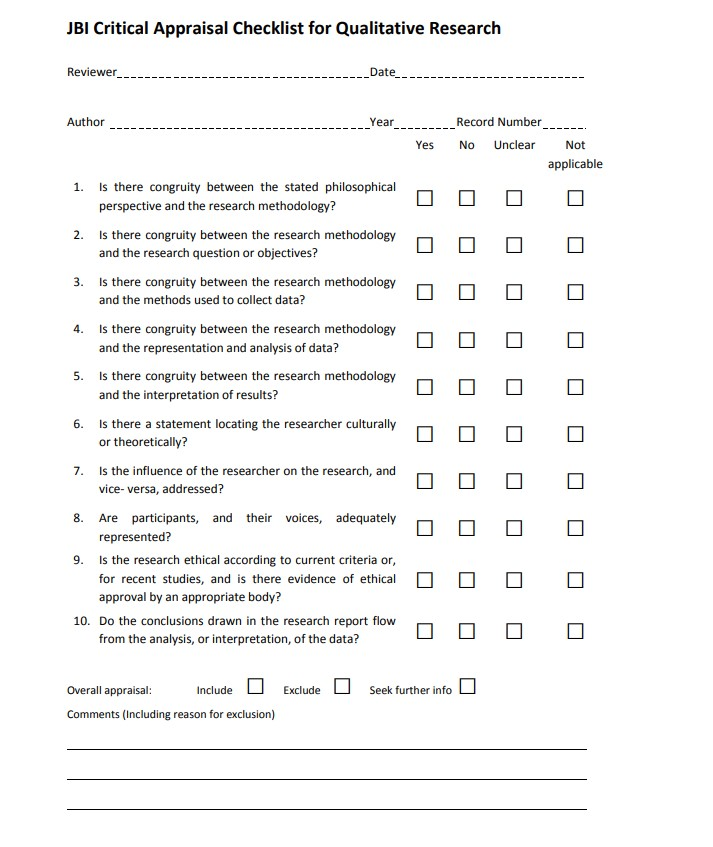


**Discussion of critical appraisal criteria**

1. **Congruity between the stated philosophical perspective and the research methodology**

Does the report clearly state the philosophical or theoretical premises on which the study is based? Does the report clearly state the methodological approach adopted on which the study is based? Is there congruence between the two? For example:

A report may state that the study adopted a critical perspective and participatory action research methodology was followed. Here there is congruence between a critical view (focusing on knowledge arising out of critique, action and reflection) and action research (an approach that focuses on firstly working with groups to reflect on issues or practices, then considering how they could be different; then acting to create a change; and finally identifying new knowledge arising out of the action taken). However, a report may state that the study adopted an interpretive perspective and used survey methodology. Here there is incongruence between an interpretive view (focusing on knowledge arising out of studying what phenomena mean to individuals or groups) and surveys (an approach that focuses on asking standard questions to a defined study population); a report may state that the study was qualitative or used qualitative methodology (such statements do not demonstrate rigour in design) or make no statement on philosophical orientation or methodology.

1. **Congruity between the research methodology and the research question or objectives**

Is the study methodology appropriate for addressing the research question? For example:
A report may state that the research question was to seek understandings of the meaning of pain in a group of people with rheumatoid arthritis and that a phenomenological approach was taken. Here, there is congruity between this question and the methodology. A report may state that the research question was to establish the effects of counselling on the severity of pain experience and that an ethnographic approach was pursued. A question that tries to establish cause-and effect cannot be addressed by using an ethnographic approach (as ethnography sets out to develop understandings of cultural practices) and thus, this would be incongruent.

1. **Congruity between the research methodology and the methods used to collect data**

Are the data collection methods appropriate to the methodology? For example:

A report may state that the study pursued a phenomenological approach and data was collected through phenomenological interviews. There is congruence between the methodology and data collection; a report may state that the study pursued a phenomenological approach and data was collected through a postal questionnaire. There is incongruence between the methodology and data collection here as phenomenology seeks to elicit rich descriptions of the experience of a phenomena that cannot be achieved through seeking written responses to standardized questions.

1. **Congruity between the research methodology and the representation and analysis of data**

Are the data analyzed and represented in ways that are congruent with the stated methodological position? For example:

A report may state that the study pursued a phenomenological approach to explore people’s experience of grief by asking participants to describe their experiences of grief. If the text generated from asking these questions is searched to establish the meaning of grief to participants, and the meanings of all participants are included in the report findings, then this represents congruity; the same report may, however, focus only on those meanings that were common to all participants and discard single reported meanings. This would not be appropriate in phenomenological work.

1. **There is congruence between the research methodology and the interpretation of results**

Are the results interpreted in ways that are appropriate to the methodology? For example:

A report may state that the study pursued a phenomenological approach to explore people’s experience of facial disfigurement and the results are used to inform practitioners about accommodating individual differences in care. There is congruence between the methodology and this approach to interpretation; a report may state that the study pursued a phenomenological approach to explore people’s experience of facial disfigurement and the results are used to generate practice checklists for assessment. There is incongruence between the methodology and this approach to interpretation as phenomenology seeks to understand the meaning of a phenomenon for the study participants and cannot be interpreted to suggest that this can be generalized to total populations to a degree where standardized assessments will have relevance across a population.

1. **Locating the researcher culturally or theoretically**

Are the beliefs and values, and their potential influence on the study declared? For example:

The researcher plays a substantial role in the qualitative research process and it is important, in appraising evidence that is generated in this way, to know the researcher’s cultural and theoretical orientation. A high quality report will include a statement that clarifies this.

1. **Influence of the researcher on the research, and vice-versa, is addressed**

Is the potential for the researcher to influence the study and for the potential of the research process itself to influence the researcher and her/his interpretations acknowledged and addressed? For example:

Is the relationship between the researcher and the study participants addressed? Does the researcher critically examine her/his own role and potential influence during data collection? Is it reported how the researcher responded to events that arose during the study?

1. **Representation of participants and their voices**

Generally, reports should provide illustrations from the data to show the basis of their conclusions and to ensure that participants are represented in the report.

1. **Ethical approval by an appropriate body**

A statement on the ethical approval process followed should be in the report.

1. **Relationship of conclusions to analysis, or interpretation of the data**

This criterion concerns the relationship between the findings reported and the views or words of study participants. In appraising a paper, appraisers seek to satisfy themselves that the conclusions drawn by the research are based on the data collected; data being the text generated through observation, interviews or other processes.

## Appendix 3: Data extraction tool for qualitative research


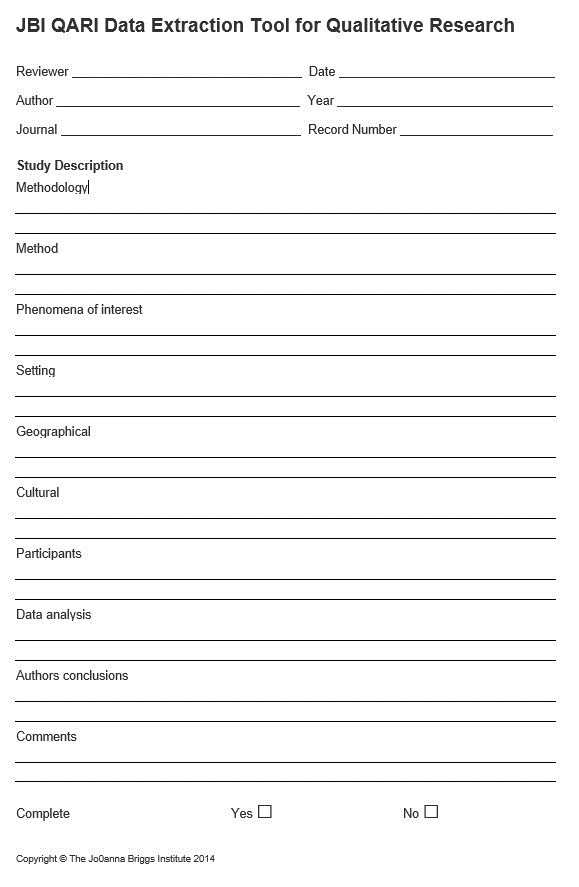


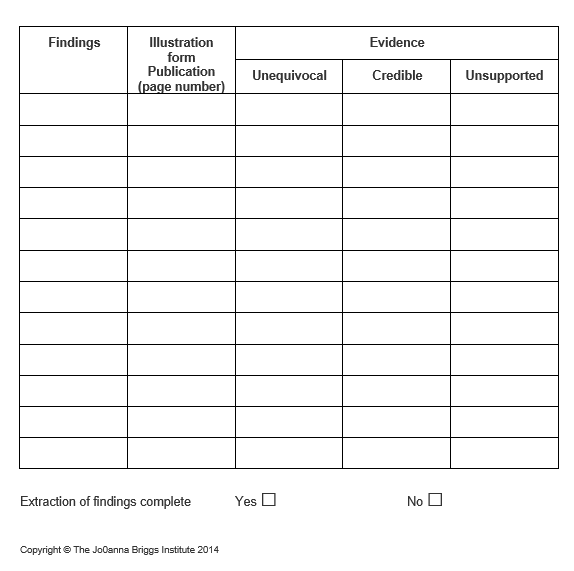


## Appendix 4: Studies excluded at full-text review

Bisogno, P. (2009). *Mental illness and recovery: A mental health support worker’s perspective: A practice research project submitted to Auckland University of Technology in partial fulfilment of the requirements for the degree of Master of Health Science in Psychology, 2009* (thesis). <https://hdl.handle.net/10292/1092>

*Reason excluded: Ineligible phenomena of interest.*

Jasper, R., Wilberforce, M., Abendstern, M., Tucker, S., & Challis, D. (2019). Support workers in community mental health teams for older people: exploring sources of satisfaction and stress. *Journal of Long-Term Care*, *0*(2019), 111. <https://doi.org/10.31389/jltc.19>

*Reason excluded: Ineligible phenomena of interest.*

Li, C., Yang, F., Yang, B. X., Chen, W., Wang, Q., Huang, H., Liu, Q., Luo, D., Wang, X. Q., & Ruan, J. (2022). Experiences and challenges faced by community mental health workers when providing care to people with mental illness: A qualitative study. *BMC Psychiatry*, *22*(1). <https://doi.org/10.1186/s12888-022-04252-z>

*Reason excluded: Ineligible participant characteristics*

Stevanovic, M., Lindholm, C., Valkeapää, T., Valkia, K., & Weiste, E. (2020). Taking a proposal seriously: Orientations to agenda and agency in Support Workers’ responses to client proposals. *Joint Decision Making in Mental Health*, 141–164. <https://doi.org/10.1007/978-3-030-43531-8_6>

*Reason excluded: Ineligible phenomena of interest.*

Sutcliffe, R. (2007). *What is the meaning of supervision for mental health support workers? A critical hermeneutic inquiry* (thesis). <https://hdl.handle.net/10292/88>

*Reason excluded: Ineligible phenomena of interest.*

Wark, A., & Gredecki, N. (2022). Proposal of a service delivery model for supported Living Community Forensic Services. *Leadership in Health Services*, *36*(1), 97–110. <https://doi.org/10.1108/lhs-03-2022-0031>

*Reason excluded: Ineligible participant characteristics.*

## Appendix 5: List of findings with credibility rating and illustrations

| Study: Screening and Referral for Postpartum Depression among Low-Income Women: A Qualitative Perspective from Community Health Workers | |
| --- | --- |
| Finding | Cultural beliefs and norms such as communication, reluctance to allow community mental health workers in the home, and reluctance to discuss mental health concerns. (U) |
| Illustration | One community health worker stated, “we deal with a lot of undocumented immigrants (and) a lot of people with different cultural diversities...(allowing)...strangers...into their home or even discussing certain things over the telephone is difficult and sometimes just taboo for some cultures...So that is a roadblock that we constantly come upon.” (P. 4) |
| Finding | Difficulties in using the depression screening tool- Community health workers reported challenges with the screening tool which sometimes resulted in either overidentification or underidentification of depression. One community health worker stated: (U) |
| Illustration | “I think that the screening tool, because...it measures (depression) within the (past) seven days...gives...(a) false number, and...makes it seems like...they are more elevated or/and they are more in need of a dire intervention because of how the questions are asked...” (P. 3) |
| Finding | Having a contact person at different mental health agencies is beneficial. One community health worker explained that this agency contact person: (C) |
| Illustration | “can expedite your call or give you information that will be beneficial for your client.” (P. 3) |
| Finding | Challenge in finding a proper approach to make referrals that are geographically convenient for the clients. One of the community health workers stated: (C) |
| Illustration | “Every client is different,…Sometimes I just clump them all together and everybody gets the same treatment…Where ever they are, you have to meet them there and try to help them rise up from it…” (P.3) |
| Study: Experiences of mental health support workers in mental healthcare practice: Three visual arts narratives | |
| Finding | Lack of clear occupational roles- Support workers are limited in their interventions by a lack of clear occupational roles. (U) |
| Illustration | "I have to say in my heart I’ve done a lot of personal growth ... I have an awful lot to offer...and I find that clients do confide in me an awful lot... but it’s only so far you can go and you gotta say ‘Well, look... go into whoever’s in the office’ ..." (p. 1024) |
| Finding | Pessimistic view of recovery -Practice staff appears to have a pessimistic view toward service user's progression or recovery making it difficult for support workers to utilize recovery-oriented knowledge and skills. Alison was confident of her knowledge and skills to support her clients however she experienced barriers in translating and enacting these practical recovery-oriented interventions among a mental health workforce that still adopts the traditional biomedical views of clinical recovery. (U) |
| Illustration | “they're going nowhere” .... “don't bother yourself.” Further, “bother(ing) yourself” seems to be actively dissuaded; “you're frowned upon for things” (P. 1024) |
| Finding | Occupational culture barriers- Support workers are not considered as part of the team and are excluded by the nursing staff resulting in a lack of collaborative practice and Sharing of knowledge between the nurses and support workers. (U) |
| Illustration | I am not part of the team... The nursing staff do not allow me in at all...and...knowing that I’ve learned so much on this course... skills, education... I’m just banging my head against this wall...and there's times I just want to give up... you know?" (P. 1024) |
| Study: Latino Community Health Workers: Meeting their Community's Emotional Needs in Intuitively Culturally Appropriate Ways | |
| Finding | Trauma and compassion fatigue- Serving people within their own community creates a feeling of emotional connectedness and leaves them feeling responsible and worried that they will not be able to meet the expectations of the people they aim to help resulting in professional stress. The participants acknowledged that their efforts to make a difference led to exhaustion. (C) |
| Illustration | “we are only a step away from being where they are.” Some have shared experiences: “I’ve been where they are, I know what it feels like to be so scared and alone.” (P. 22) |
| Finding | Safety at work- Some participants reported to have been challenged and attacked by community members. They reported feeling unsafe and worried. They reported being told that they were providing care to: (C) |
| Illustration | “illegal aliens.” and felt “singled out” (P. 22) |
| Finding | Culture insensitivity- Support workers feel that they are left to mend medical professionals’ neglect, insensitivity, and ignorance in regard to culture.They explain cultural nuances to medical practitioners, and also help consumers understand the cultural context from which the practitioner communicates. They reported that they were used like: (C) |
| Illustration | “cultural brokers.” (P. 21) |
| Finding | Lack of Support and supervision from the administrators and Supervisors. Participants requested for training of the administrators and to be supported through supervision. (U) |
| Illustration | “Lack of understanding from administration and supervisors” who “think our job is simply providing information. They do not understand.” (P. 22) |
| Finding | Lack of support from co-workers- participants reported that some workers who were not of Latino heritage failed to recognise their work.. (U) |
| Illustration | “It is hard enough that we do so much. When our coworkers are not supportive, we have to do double the work.” (P. 22) |
| Study: Supporting recovery: challenges for in-home psychiatric support workers | |
| Finding | Care provision versus promoting autonomy- Support workers found it challenging to balance providing care for a severe and disabling illness and promoting independence. The challenge was mainly in the client's participation in community activities and completing household chores. Regarding housework, Support workers reported that their role was to promote and encourage their clients to complete their housekeeping to develop a necessary life skill. The client's home was also viewed as a support worker's workplace that was required to be safe. The following illustration describes the views of many support workers: (U) |
| Illustration | "[With regards to cleaning] I guess “assisting” is what we’d mainly be doing. The idea being to motivate them to be doing things independently themselves [y] if someone’s capable of doing the dishes, it’s not that we don’t want to help them. But especially with the medication sometimes, they use that as a bit of an excuse – “I’m lethargic and tired”. So you could easily be doing everything while they sit back and watch TV, so that’s not what part of our job’s about. So we’ll assist them and encourage them and motivate them in any way we can." (P. 76) |
| Finding | Encouraging clients to meet their goals- Support workers encouraged their clients to set goals as part of inspiring hope. However, they reported challenges in encouraging goal-oriented behaviour due to the fluctuating nature of their client's illness. As such, they needed to be flexible and understanding when their clients did not actively engage in activities to achieve their goals. One support worker said: (U) |
| Illustration | "So a person that we work with can articulate their goal and say this is really what I want to achieve. And we can have all the things in place to do that, but if they then decide “Well I don’t want to do that”, then what we have to do is then give them space, there’s no way we can force that [y] you have to be prepared to be flexible. This particular line of work requires flexibility, you sort of go with the moment. Sometimes it can be just a pure babysitting role, that’s what we’re doing. Other times we are just powering on to achieve things. So I would say some of the barriers just personally is just being able to be patient and be flexible to allow things to take their natural course ." (P.76) |
| Finding | Boundaries between being a friend and a worker- Establishing a therapeutic rapport with clients is crucial for the support work role. However, it is challenging for the support worker to establish a therapeutic connection within the client's home or the community as the support worker may be viewed as a friend by the client in comparison to workers establishing relationships in other settings such as clinical areas. Support aims to promote independence and encourage clients to integrate into the community. As such, when a support worker fulfils the need for friendship with their client, they are likely to lower the client's motivation to develop "natural" relationships. One support worker stated the following: (U) |
| Illustration | "I think one of the problems with the service we’re providing is that in some ways we become their friends. We are not, but as part of the role we do provide a level of contact that can be articulated as friendship to them. So some of them identify us as friends; we’re not just workers, we’re friends. And I guess by doing that [y] that probably stops them from looking outside for friendship." (P. 77) |
| Finding | Supporting an aggressive client- Supporting clients with behaviour was difficult for the support workers. One of the participants stated the following regarding supporting an aggressive client: (U) |
| Illustration | "[y] that particular client, he is not very stable [y] I’m in the process of educating him about it. It’s a sort of a process that I don’t really like doing because it involves me making some tough decisions for the client but sometimes these decisions have to be made in order for him to understand the consequences of behaviours, like you know if you’re going to abuse me and if you’re going to be angry I don’t want to be around you. And nobody will want to be around you, and you’re not going to get friends if you keep like this [y]." (P.77) |
| Finding | Promoting citizenship- Support workers reported that it was challenging to encourage clients to participate in community activities. (U) |
| Illustration | "So I’ve sort of come to the conclusion that if they don’t want to do anything like that [participate in the community], well that’s fine. If they just want to go for a walk or watch TV, or whatever, that’s their quality of life. That’s what they want to do." (P. 76) |
| Finding | Close relationship with clients- Having a close relationship with clients was seen as a facilitator for the support work role. One of the support workers stated that she enjoyed the close relationship she had established with her clients over the years. (U) |
| Illustration | "I just [y] like them. I like, they’re all just unique and special and they’re just so nice. And they’re so thankful, and they’re so appreciative of the support. And the fact that, a lot of them now, they still see me as a support worker, but as a friend as well. And they just appreciate the fact that someone wants to give them the time of day. And I think that’s a major thing. I’ve had clients of mine come over for Christmas day and things like that." (P.77) |
| Finding | Respecting client's autonomy- Some younger clients had a drug and alcohol problem. Regarding this, the Support workers reported that it was challenging to balance between respecting the client's autonomy and their perspectives of what constitutes a healthy lifestyle choice. (U) |
| Illustration | "We’ve had to say that we will not purchase alcohol because that was becoming a concern. We’ll take them to a club or a pub, and quite comfortable for them to have a couple of [drinks], but not to the Bottle-O to purchase alcohol. We will drop them in the vicinity, you know that’s what they’re going to do, but they can find their own way home." (P. 78) |
| Study: Training for in-home psychiatric disability support workers | |
| Finding | Motivating clients- This was identified as a significant challenge by most support workers. One of the support workers expressed the following: (U) |
| Illustration | "Just some of the ways of helping us, because some of them are on such heavy medication it’s not always easy to motivate them, so maybe some sort of training in, not necessarily goal setting, but working with the medication and towards maybe achieving their goals within their capacity. . .Because a lot of them do, with the medication, lack that motivation.” (P. 40) |
| Finding | Knowledge about symptoms of mental illness and medications- Support workers spend the most time with the clients. Thus, they are better placed to identify any problems related to symptoms, medication, or physical health of their clients. However, identifying these problems is difficult for the support workers because they do not have adequate knowledge. One of the support workers indicated that they would like more training in: (U) |
| Illustration | "Medication. Learning the medications, their side effects, what they actually are for. I think that would benefit a lot. Because we’re told we’re supposed to look for signs of physical health that could be caused by medications but sometime we won’t know. Because we don’t know what they’re taking." (P. 39) |
| Finding | Chronic nature of clients' illness- The chronic nature of the client's condition, its effect on them and extended periods of institutionalisation made it difficult for support workers to apply recovery principles. The following are the views expressed by one of the support workers regarding working with a client who had a long history of institutional living: (U) |
| Illustration | "P300 clients I support generally want to maintain their current lifestyle and routines, they express dreams and wishes but rarely take any steps towards these as a result of many past failures and disappointment. This makes recovery difficult to apply." (P. 40) |
| Finding | Challenging behaviour- This was mainly indicated in some of the written comments and by one of the support workers in the interview. The following are the views of the interviewee and some of the written words: (U) |
| Illustration | " . . . But certainly it can be really challenging if someone is really angry or abusive to you and stuff like that. That happens now and then and for me that might be one of the challenging times . . ." (P.40) ‘‘behaviour in home and public sometimes’’, ‘‘challenging behaviours – non-compliance, insight issues’’ and ‘‘when my client refuses to do anything while you support him’’. (P. 40) |
| Finding | Impact of long-term illness and institutionalisation- another support worker mirrored the sentiments about the effect of institutional living by saying: (U) |
| Illustration | ". . . I think that some of the clients definitely have institutionalised behaviour. You know, like they like their routine, they like things done a certain way. But recovery for them might not mean, I don’t know, it’s like, just being able to give them the freedom, and be more spontaneous and flexible with the things they do ...." (P. 40) |
| Study: Negotiating the boundaries between clinical and non-clinical work within a supported housing program | |
| Finding | Poor communication- There was no regular communication between the case managers and the support workers. One support worker stated the following: (U) |
| Illustration | "And another [client] I’ve been working with for 41/2 years and never knew he had [a case manager] … the reason she gave me on the phone for why she hasn’t really spoken to me before was that “you guys have so much input into him and he’s going really well. I haven’t had to come in very much.” But on the other hand, we really need to have a bit more communication with people like her on a regular basis so she does know what’s going on." (P. 225) |
| Finding | Unclear role boundaries- support workers felt that sometimes the case managers expected them to perform clinical tasks such as monitoring medications. In such situations, the support workers would affirm their role limits. One support worker gave the following illustration: (U) |
| Illustration | "I said to [case manager]“you do this part, we do that part, you’re the clinical partand we’re non-clinical”...Sometimes they [the case managers]...were almosttreating our staff like they were clinical staff and that’s a problem...it’s like“we arenot clinicians, and we’ll never be...that’s not something we aspire to be." (P. 224) |
| Finding | Frequent change of case managers- the clients' nominated case managers changed frequently. Support workers reported difficulty in locating new case managers when a client was showing signs of deterioration. Additionally, for clients who had ceased being case-managed, it was difficult for support workers to access the required support necessary for determining whether such clients needed extra mental health care. The following is an illustration from one of the support workers: (U) |
| Illustration | "There are many clients without a case manager … and to me, that means that I myself or my coordinator has to make a decision about whether [the client] needs extra care or not, and I don’t think that’s good enough. We’re not medically trained … We need a backup." (P. 225) |
| Finding | Difficulty accessing clinical expertise- Once clients seemed like they had settled into their homes, case managers appeared to disengage from them. Support workers reported that it was difficult to contact the case managers as illustrated by the following: (C) |
| Illustration | "I would say through my experience over the years, contact to the mental health services is a one-way street. I would say out of every 100 phone calls, 99 of them would be made from here [support agency] to there, not the reverse. No matter what the scenario, no matter what the request." (P. 225) |
| Study: An extra pair of hands? A case study of the introduction of support workers in community mental health teams for older adults | |
| Finding | Health professionals influence- Support workers reported that their input was highly influenced by the professional practitioners' understanding of the support work role. They felt underutilized because their contribution to the team was usually neglected. Team 2 support worker stated the following: (U) |
| Illustration | "When I started it was really like (clinicians)defaulting to what people used to do. And when I felt I could help, I've tried to change that, but you have to be really assertive and really push...it's like jumping around saying ‘‘I'm here, I can do that.’’ (P. 738) |
| Finding | Lack of awareness of support workers role- Support workers complained that some of the team members were not aware of support workers' functions within the team. Support worker from team 4 stated the following: (C) |
| Illustration | "The different ideas within the team...that sometimes felt like a battle, or sometimes a bit demoralising." (P. 737) |
| Finding | Work assignment- Support workers reported being considered to be more of a health care assistant and as such their full potential was not realised by other team members. Team 1 support worker stated the following: (U) |
| Illustration | " I think some of my colleagues did feel that I was more of a health care assistant." (P. 738) |
| Finding | Lack of role clarity- The sentiments of the group 4 support worker were mirrored by the group 2 support worker who stated the following: (U) |
| Illustration | "I would have liked to just have a clear defined job and not been so frustrated trying to convince people what I should be doing. " (p.737) |
| Finding | Limited training opportunities- Support workers reported that there were not many training opportunities for them. The team 3 support worker stated the following: (U) |
| Illustration | "It is very good experience for me, but one thing I've found is there's not much training out there for support workers, to actually improve ourselves, except than people go and study other professions like OT or nursing. There should be something there for a support worker to carry on up the stepladder" (P. 739) |
| Finding | Role ambiguity- Group 3 support worker also raised concerns related to role ambiguity by stating the following: (U) |
| Illustration | "The problem is the CPNs in my team are not sure about my role; they still don't know what I'm supposed to do, even now." (P. 737) |
| Study: Beyond formalized plans: User involvement in support in daily living - users' and support workers' experiences | |
| Finding | Limitation of written support plan- The support work role should be flexible depending on the presenting situation and should not follow setup rules rigidly. Formal plans were seen as a long-term goal rather than detailed instruction. While the support plan is written in consultation with the service user, it does not take into consideration the changes in the user's everyday life. Tommy who was one of the support workers stated the following: (U) |
| Illustration | "To not exactly follow regulations that someone else has formulated. Because I know what I work with, I know what to do. I do not always need someone who has sat behind a desk and made a plan that this is the way it will be." (P. 158) |
| Study: Recovery, non-profit organisations and mental health services: ‘Hit and miss’ or ‘dump and run’? | |
| Finding | Community understanding of recovery- The participants reported that the community's understanding of recovery was also different as illustrated by the following statement from the focus group 2: (U) |
| Illustration | "[There is a] picture coming from above that recovery means integrating people … into the mainstream community. But the reality is that our client base actually has a community here … that’s not necessarily connected [to] traditional ways with nine to five jobs, and mortgages, and … white picket fences … things the mainstream community seems to think everyone is aiming for …’" ( P. 356) |
| Finding | Bureaucratic requirements of the government-funded health service organisation- while a written recovery plan ensured consistency in care it did not allow choices that were adaptable to individual consumers changing needs. Additionally, it limited the care and support provided by the community workers. (U) |
| Illustration | "… this recovery plan with specific boxes that you need to complete, it’s challenging because you need a system, but at the same time you need flexibility and choice that is adaptable to individual people … It cannot work the same for the rest of their life, or our lives; it has to change according to the needs and basically the demands of life" (Focus group 2) ( P. 356) "… this comes back to your bureaucracy thing, “No, that ain’t in my box, it’s not my job, I don’t deal with that.” Well people are complex and big, and there’s lots of stuff going on that doesn’t necessarily fit neatly into one section" ( Focus group 2) ( P. 357) |
| Finding | Common aim- Having a common aim between the clinicians and community workers including the delivery of consumer-centred care to people with mental illness was beneficial in ensuring that the care provided was consistent. Having regular meetings, joint plans as well and monthly meetings facilitated better collaboration between the clinicians and the community workers. A participant in focus group 1 stated the following: (U) |
| Illustration | "We seem to be working collaboratively better, having joint [Recovery] plans … and we also regularly keep [the clinicians] informed with monthly reports, and regular meetings … there seems to be a more common focus of moving towards recovery … working with people to try and improve their lives, I think there’s a growing understanding of what we’re all trying to do, that we’re all on the same side". (P. 355) |
| Finding | Understanding of the role of community workers by the clinicians- Clinicians viewed community workers as having a very limited role in the implementation of recovery-oriented care. The Participants reported that they had to negotiate and make suggestions to clinical managers about what they could do as community workers. A participant in focus group 1 stated the following: (U) |
| Illustration | "In the early days we worked with many clinicians and they saw how Recovery [works] and what we could do as a community organisation was very different to what they thought we could do. They saw us as having a very limited role, and over a period of time there’s been a lot of conversations with clinical managers, a lot of suggestions – well maybe we could do this, and then there would be agreement, yes – you can do that". (P. 355) |
| Finding | Different understanding of recovery among clinicians and community workers- Clinicians and community workers had different understandings of recovery care. It was challenging for community workers when their input toward consumers' recovery was disregarded by clinicians. A participant in focus group 1 stated the following: (U) |
| Illustration | "I came in with a view of recovery that has lots of possibilities, and I still hold that for people. [But] I think probably that’s … one of the things that I come up against fairly regularly – the vision that a clinician might hold for someone and what I hold for someone can be quite different. [But] I think I’ve been tempered a little bit. It’s challenging to have my ideas of where someone might be able to go in their recovery kind of squashed, or seen as childish, or not possible, so that’s been interesting to sort of play around with that. At the same time I’ve been educated by the clinicians about [something] that may not be possible and [so I] might have to accept that for the person. So I’ve needed to be tempered a bit as well in my perspective." (P. 356) |
| Finding | Lack of communication between the clinicians and community workers- Participants reported that communication between the clinicians and community workers was a challenge. When a consumer was discharged from the hospital, the community workers were not handed over information about the consumer. A participant in focus group 1 stated that sometimes they were not notified of the consumer's discharge. (U) |
| Illustration | "… at discharge which is a critical point, someone might have [a NPO community worker] … for 18months or a year. The worker is not even told that they’re discharged, like a family member may not be told" ( P. 356) |
| Finding | Building individual and service relationships- Building a service relationship was identified to be challenging in comparison to building relationships with individual clinicians. (U) |
| Illustration | "[There is a] challenge between building individual relationships and service relationships. Individual relationships we’ve found easy to build, or certainly in my experience; you know, you find a good clinician, you’re working with a [long term] consumer, you can build good relationships. But the service relationship is more challenging to build, which is a problem because if the system is working it shouldn’t matter who’s delivering [the service." ( focus group 2)( P. 357) |
| Finding | Good relationship with clinicians- Having a two-way conversation between the clinicians and the community workers enhanced their collaboration and facilitated learning from each other. A participant in focus group 1 stated the following: (U) |
| Illustration | "It’s been a two-way conversation actually and the more we can do this … the better it works on an individual basis with clinicians where there’s been an openness to learn from each other. In this year and a half, we’ve come across plenty of clinical managers who we’ve established relationships with, where we both have actually … [been] willing to listen and learn". (P. 355) |
| Finding | Formal agreement- Having a formal agreement between one of the non-profit organisations and the clinical mental health service enhanced the collaboration between the two organisations resulting in better outcomes of the care provided. The following is an illustration from focus group 1: (U) |
| Illustration | ‘It’s changing. Before [the implementation of Recovery-oriented services], I never saw a recovery plan. There was a recovery plan by clinicians and sometimes they did these with the client, but we as community workers, no-one was bothered to show us [the] recovery plan. So now it’s changing, maybe specifically for our program because we do have partnership by agreement with mental health – we have to see and we have to have this recovery plan, but I think as far as for other services, we don’t have these same partnerships, they receive at least a recovery plan, even if it’s not an ideal recovery plan, at least we now have some kind of [a plan] …’ (p. 355) |
| Finding | Clinicians lack of understanding of the role of community workers- Establishing a good rapport between the community workers and clinicians was challenging. The participants felt that the clinicians did not understand the role of community workers as shown by the following illustration from focus group 1 (U) |
| Illustration | "We’ve absolutely had to face off and have difficult conversations and managed the interactions that can be quite [challenging?] … there could be attitude coming from both sides. We have to work at our attitude and our listening up and their listening up to us, so we’ve actually had to relate, we’ve had to learn to relate. And at the director level that’s been very difficult and we’ve had to learn to do that at the director level, and that was really obvious that it was like … we want to be respected in terms of the work that we’re providing in the community, but we weren’t getting the respect. It was not in the picture, so that was something we had to keep working at, keep fronting up, keep sort of … standing up for that, keep saying what we’re doing, chipping away, chipping away and then just recently that’s sort of like … phew, we’ve actually got to something in terms of that relationship." ( P. 357) |
| Finding | Clinicians caseload- The high caseload of clinicians was perceived as a hindrance to information sharing resulting in what a participant in focus group 2 referred to as a "dump and run" situation following a client's discharge. (U) |
| Illustration | ‘… there’s a few clinicians that I would speak really highly of, and I think do a really good job, if you can build a rapport with them and the client wants you to go with them and things like that. But the majority of my interactions, I feel like it’s very dump and run, “You look after them, I don’t need to see them. I’ll just see them if there’s a problem.” That’s my experience’. (p. 356) |
| Finding | Frequent change of clinicians- high turnover of clinicians was reported to impact the collaboration between community workers and the clinicians. Having the same clinician for an extended period of time resulted in better collaboration and outcomes for the consumers. One of the participants in focus group 2 stated the following: (U) |
| Illustration | "It’s really good when it’s good, but my experience generally with a lot of the [clinicians, is that there is a] high turnover … With one of our clients, who I’ve been working with for a year, we had the same psychiatrist and clinical manager for the whole year … and there was a really strong sense of collaboration, and there’s been, well, fairly good improvements for the client actually, so I found it very consistent. But that’s just, yeah, with one client." ( P. 357) |
| Finding | Poor communication between the community workers and clinicians - communication and sharing of information between the community workers and clinicians was a major challenge. A participant in focus group 3 stated the following in regard to communication: (U) |
| Illustration | ‘It’s a bit of hit and miss. But if [we] keep up the communication, keep up the pushing of it, it [does] happen. Since [the implementation of Recovery-oriented services] there’s been a shift … By really trying to push and work collaboratively, it does work with individual [clinicians]’. (P. 357) |
| Finding | Lack of understanding of community work role- Clinicians did not understand the role of community workers. Participants stated that some of the clinicians were not aware of the existence of their services. a participant in focus group 2 stated the following: (U) |
| Illustration | ‘And also even, you know I’ve personally done a lot of promoting our service to the public health services, like I’ve gone out and spoken to the regional deliverers … and interfaced and said, “This is what our service is,” but I still meet plenty of clinicians within the public health service that wouldn’t have a clue that we exist – any of our services’. (Focus Group 2) |
| Finding | Common understanding of goals- Shared goals were important in ensuring the provision of holistic care to consumers. The following are illustrations from focus groups 1 and 2: (U) |
| Illustration | "Before … I never saw a recovery plan … No-one was bothered to show us … [but] now it’s changing …". (Focus Group 1) "Now … the planning has actually improved … we’re actually looking at what are the shared goals that the service and the public health service can work towards for this person, and it is consumer driven. So instead of looking diagnostically in a medical model at what’s wrong with them, and how do we deliver a therapy, it’s about, “Where do you want to go and how do we help you get there” and being on the same page". ( Focus Group 2) ( P. 355) |
| Finding | Disregard of support workers care input - Participants reported that their input was dismissed by the clinicians especially when they tried to advocate for the consumers to the clinicians. This was attributed to the participants' lack of tertiary-level qualifications. A participant in focus group 4 stated the following. (U) |
| Illustration | ‘I have had other colleagues here say they’ve wanted medication reviews for their clients and the doctors pretty much brush[ed] them off because they think we’re just support workers’. (P. 357) |
| Finding | Different approach to supporting recovery- Following a written template ( recovery plan) was reported to limit the support that the community workers provided to the consumers. A participant in focus group 1 stated the following: (U) |
| Illustration | "What’s been useful in the roll-out of recovery is they have one sort of format/template that they’re working with, although for us that can be a little bit limiting because not everybody relates to a piece of paper with boxes on it." ( P. 356) |
| Finding | Relationship between the community workers and the clinicians- The relationship between the clinicians and the community workers was described as "hit and miss" more than once by the participants. While they were supposed to work as partners the community workers felt that they were not considered part of the partnership. The following are illustrations from focus group 1: (U) |
| Illustration | "… engagement between non-government organisations (and the health service) is quite hit and miss" (Focus Group 1) "We’re supposed to work in partnership to be on the same side but sometimes I have the feeling that we are on another side." (Focus Group 1) ( P. 357) |
| Finding | Consumer understanding of recovery- Community workers found it difficult to explain what recovery means to some of the consumers, especially to consumers from different cultural and language backgrounds. Some consumers were also frightened by the written recovery plan that the community workers were required to complete. The following is an illustration from focus group 1: (U) |
| Illustration | "In the community sector … [we] relate to different visual cues or different ways of even describing recovery. For some people, “recovery” doesn’t mean anything … so [the journey] is about how you get to that place of looking at what’s hopeful and what that person wants to build or work on, so it may not be called recovery, but that’s what we call it, but that word may mean nothing to the person that we’re working with … Especially if you have people from a different cultural background or coming from a different language background. It’s very hard to explain what recovery means or … what is my role or what is the clinician’s role and then you come up with a piece of paper that is called a recovery plan and you have to fill out the boxes. For some people … [this is] frightening" ( P. 356) |
| Finding | Rigid care plans- While having a recovery plan was beneficial, it was not flexible to accommodate the changes that are constantly happening in consumers' recovery process. The following is an illustration from a participant in the focus group 2: (U) |
| Illustration | "It is … I feel recovery is constantly changing. Everyone’s in recovery for something. Like you can’t just say, “Here’s your recovery plan, we’ll check it out in three months, or six months, or whatever.” I just don’t think it works like that, you can’t just write things down and go, “Yeah, check that out.” (P. 356) |
| Finding | Support work role acknowledgement by clinicians- The participant expressed the need to have their role acknowledged and respected by the clinicians as equal providers of recovery-oriented services. The following are sentiments of 2 participants: (U) |
| Illustration | "… we want to be respected in terms of the work that we’re providing in the community." (Focus Group 1) "I also wanted to point out that if they say we’re going to work together, they should treat us as equal. I don’t feel that they treat the community sector workers as something on their level; they think we are inferior to them. Yeah. Because maybe they have more clinical knowledge than us, but I don’t know about their other knowledge in other areas, because I’ve seen them, sometimes they talk rudely, or sometimes they don’t know how to talk, or how to approach, and they try to avoid the situation … So I’ve had this feeling that they don’t treat the community sector workers as something important." (Focus Group 2) ( P. 357) |
| Finding | Being viewed as transport service providers- Participants reported being seen as the consumers' transport service providers by the clinicians. Participants in focus group 3 stated the following: (U) |
| Illustration | "I still … come across pockets of one or two, more so with higher level health professionals who may see us as more like … We’re not seen as clinicians, and we’re not seen as being a major player or stakeholder, we’re more or less maybe a transport service … And that’s one thing we’re trying to change, is some doctors have seen us as a transport service, to pick them up and drop them off for medical visits or so forth. So we’re trying to say, “No, we’re more than that.”’ (Focus Group 3) "We are mental health recovery workers, here to provide a service and a Recovery-orientated approach. We are not just a taxi." (Focus Group 3) (p. 357) |
| Finding | Difficulties obtaining information- Participants had difficulties obtaining information from some of the clinicians despite making multiple requests. The following are the views of participants in focus group 2: (U) |
| Illustration | "[You may find] one clinician and work with them, and they would have a recovery plan for somebody. They’d give that to you, they’d do regular [Life Skills Profiles], and give those to you … And then you’d have another three clinicians who would say to you, “A recovery … what?!” … They’ve never even seen [a recovery plan]! Or you’ll ask them [for a recovery plan] and they’ll say, “Yeah, I’ll send that to you” and six months later you’ve asked 50 times, you’ve sent a hundred emails, you’ve rung them, you’ve begged, you’ve pleaded, you’ve grovelled in the dirt … and you just don’t get anything …" "I’ve seen that problem with many health professionals. You keep emailing them, keep asking them questions, but there is never an answer" (P. 357) |
| Finding | Being viewed as companions- Participants reported being viewed as consumers' companions. They stated that their effort to share information that was relevant to the recovery process of the consumer was disregarded by the clinicians. A participant in focus group 2 stated the following: (U) |
| Illustration | "[The clinicians] just seem to treat us like we’re a bit extra, that we are not qualified or anything. We’re just there to hold people’s hands while they do their bit, and we are just … [pause] … Yet we are the ones who see [our clients] on a regular basis … and we can pass on some very important information to them, but we basically get, “No, you’re just case workers.” It’s like a qualification snobbery hierarchy." ( P.357) |
| Study: Who cares about carers? Experiences of community mental health support workers from a feminist perspective | |
| Finding | Need for better pay- Participants reported low wages compared to their job responsibilities, risks, challenges, and relative to other professions. (U) |
| Illustration | “I definitely think the work deserves to be paid better. There are bits in the role which can be very low skilled, just driving from A to B but at the same time there are bits which you really do to need to be on your game, noticing signs or just when someone has had a bad day and that, you do need to be able to help them through that, stressing out or something like that. Every so often we get assaults, not necessarily at us but just somebody assaults somebody else and we are involved with one or more of the parties involved. Or people get suicidal and things like that so you do have to be able to cope with that and react appropriately and for that I think, I think it does definitely deserve more financial recognition”. (P. 74) |
| Finding | Working in isolated and dangerous situations- Some participants reported that their colleagues had been sent to work alone in isolated and dangerous situations with no cell phone coverage and no way to call for help. (U) |
| Illustration | “For example one of my colleagues… she got sent up to see a guy that was completely psychotic and when she got into the house she found out that she couldn’t actually get out, and the other thing was that the house was based in the country side and there was no phone coverage”. “There was only really one particular time where the client was quite suicidal and they were remote and there was very little cell phone coverage and I couldn’t get in touch with anybody and in that situation you just feel really helpless. Basically I called an ambulance and got them out, with what little cell phone reception I could find. The client was definitely in danger and they seemed to be very physically unwell”. (P.101) |
| Finding | Limited resources to support staff safety- Some participants reported that there was limited funding and resources to enhance security when working with high-risk clients or clients who have a history of assaulting staff. (U) |
| Illustration | “The funding cuts are affecting our safety. A good example is when this client was coming back…we held some meetings and we involved the union like I said, and we had a list of the things we wanted, some of it included some training, we wanted radios, we wanted a behavioural plan made up like a flow chart, we wanted a clear glass put in the office door so we could see, because this client will just sit there and knock, knock, knock, knock on the door, so we wanted a clear piece of glass so we could communicate to them through the door, without having to unlock the door, and so they promised all of this stuff and some other stuff, and that it would all be done, but the reality was that none of it got done before the client had arrived” ( P. 103 - 104) |
| Finding | Staff safety- One of the participants said that their supervisor ensured their safety. (U) |
| Illustration | “Usually our supervisor will scout out the places first as well, if we are ever going some place new we sort of have some brief as to what the layout of the land is going to be, the cell phone reception, whether there is steps that are broken or if its dark or anything like that, so we know this is not the sort of place I go to by myself or at night, or I don’t go inside, so we can establish those boundaries very easily” (P. 101) |
| Finding | Inconsistent care delivery - Inconsistency in the way staff approach their work with clients was reported to be a challenge by some of the participants. (U) |
| Illustration | “Its interesting because the diversity of the staff and also the nature of the role, there are quite a few staff having just come on board recently, I find that there is a little bit of variation in how people go about their work or tell you what you are supposed to be doing.” “The clients would get moved around and work with different staff, so the clients had different expectations of staff as different staff had different personal boundaries and so it would be quite challenging to begin working with a client who had been with a support worker with very little boundaries and for them to accept working with a staff member with much clearer boundaries”. (P. 98) |
| Finding | Verbal and physical abuse from clients - Participants reported challenges with abuse from their clients. Verbal abuse was said to be common in mental health support work. Some participants had experienced numerous occasions of assault and threatening behaviour from clients. (U) |
| Illustration | “…one of the residents in the house has quite a volcanic personality and will maybe throw things around a bit and stuff, not necessarily really at people but that has no doubt happened too, but that is few and far between and that’s I guess what you are there for.” (P. 82) “...like a lot of people we support, they have been brought up in a environment where there wasn’t any hope for them to start with and so they have been introduced to drugs and violence from a very, very young age through their parents and so now when you are trying to negotiate or reason, it can be very difficult to get any message through and there are a lot of assaults and that going on, a lot of abuse a lot of shouting, a lot of swearing and some days you might have some small break throughs and get what you are trying to get and other days its just like hitting a brick wall”. (P. 83) |
| Finding | Different approaches to care delivery- Some staff were reported to utilise a condescending approach to their support provision, which impacted the effectiveness of the support provided. (U) |
| Illustration | “…it’s the gut reaction you get when you see someone that really needs your help and you just want to help them and do everything you can for them and I think that’s what it was and these clients would be with the organisation for a long, long time say up to 4 years, because they just weren’t having that support to go out and do their own stuff, whereas the clients I would work with and staff with a similar approach, we might only see them for five weeks for example and then they would be off, they have a got a job and they would be doing their own thing”. (P. 99) |
| Finding | Lack of support from the managers. (U) |
| Illustration | “…she wasn’t very good at caring for her staff members per se and I realise the extent of that now when I started in a new organisation because where I work now for instance, if something went wrong or if she heard about it the first thing she would do when she rings me is say ‘are you ok?’ and that’s just amazing because my previous manager would be ‘what have you done’.” “There’s just a lack of care from management, that’s what it is, it’s as if they just don’t care. ‘We’ll we’ve got the budget, just do your job, we don’t care what kind of job you do, just do it, and well get our budget next year and carry on’.” (P. 109) |
| Finding | Lack of structure around wage increase (U) |
| Illustration | “Pay varied between staff members… in my current job I started on lower pay but I managed to climb up consistently, systematically, they said these are the steps, this is what you need to do, this how you go up. In mental health support work it was very unclear, so that was very unrewarding.” “…at a previous employment of mine we had an annual increment that you went up as a matter of course and you also had CPI adjusted increase every year, my organisation doesn’t have anything like that, you might get an increase but its not a given, you don’t automatically move up, you have to go through this performance review thing”. (P. 75) |
| Finding | Ongoing training - Some participants reported receiving ongoing training was beneficial to their role. (U) |
| Illustration | “The training is actually fantastic, really good training, ranging from stuff like CPI stuff which is not quite a martial art but how to deal with potentially volatile situations, how to notice when they might occur and what to do afterwards all the way through to cultural training to suicide prevention training to things on autism, or more specialist sort to of areas, we usually get several days worth of training each year”. (P. 96) |
| Finding | Lack of appreciation by the management- Participants expressed feelings of needing to be more valued and appreciated by the managers. (U) |
| Illustration | “My manager gave me no appreciation” “No there is never any recognition it. There is no ‘how are you feeling, that must have been quite hard’. But the team, what we will do, there might just be a pat on the back, and it’s just an acknowledgement, ‘hey I know what you’re going through’, or it may be ‘do you want to talk, do you want to sit down and have a cup of tea?’, yeah so we are good like that but we don’t get that from management, we don’t get management coming up and saying you handled that really well or anything like that.“…more support rather than being criticised would be pretty nice, especially for more experienced staff like us you know, even for new staff, it’s just not nice, I know they have a contract with the DHB and they have a hard line to play, I suppose to keep, as our manager says, they are our bread and butter, but even so there is still a way to treat people with respect and dignity, and when it’s not been happening its pretty clear and obvious”. (P. 105 - 106) |
| Finding | Lack of proper supervision and training to deal with challenging situations- Some participants reported feeling emotionally drained from working with people with chronic mental illness without appropriate training and support. (U) |
| Illustration | “There would be some clients that were just so, so heavily depressed or had so many issues that you would get dragged into that stuff and being quite naïve about the whole thing and not having the proper supervision or training, that just happens and it’s really difficult because you get brought into all their emotional issues and it really affects you and so that can be really difficult and completely emotionally draining.” “It took a year working in the job to shut out work at home. That’s quite normal I learnt with other people. You end up mentally taking your work home. And that’s not until you get a grip, because it takes about three months to get used to a new job, but the mental element with mental health just took a lot longer, but once you learn to shut it off and not to worry then it becomes a lot easier, but I do remember that being quite difficult for at least a year”. (P. 84) |
| Finding | Support workers opinion not valued- Participants reported that their opinion was not valued by clinicians. (U) |
| Illustration | “…if you’re not a psychiatrist or an intellectual as far as they are concerned, then you’re not really worth talking to at any sort of valuable level.” “..it is not a valued job, it is not a valued role. Because I was thinking… I will do this job for quite some time, even though there is no status involved and there is not an increase in pay and I get treated like shit on the hierarchy as far as being in my role, because there are people who won’t even look at you if you are not as high up as they are or even talk to you because they are just too superior, because they are doctors or whatever but I just figure, I was thinking, being able to do this job, I can do more to help people in this job I think than in a lot of other jobs”. (P. 108) |
| Finding | Staff safety and well-being- Some participants reported that their work can be psychologically draining and unpleasant; however, the managers would not allow them to take a break in such situations. (U) |
| Illustration | “…the client just went off and were screaming at me and abusing me and then they rang the crisis team to tell them to come and get me… and this went on for probably an hour, where they just stood at the locked door and just screamed at me… by the end of it your really just fatigued… now that can happen through the day that sort of behaviour…so it would be really nice just to be able to go out for a walk or to jump in the car and go down to the water for half an hour, it would be really nice to do that but of course we can’t do that. So that verbal abuse is really common in our house because there are several clients that will talk to you like that.” (P. 103) |
| Finding | Lack of job and pay progression- Participants attributed job dissatisfaction and high staff turnover to poor working conditions and lack of pay advance. (U) |
| Illustration | “…it’s considered a dead end job and this is as far as you go with this qualification which is level five, you can get in from either doing 2 years of nursing training or doing level five certificate and I think that again this is feeding into the expectation or stereotype that people who do mental health support work haven’t got the brains or the knowhow or the motivation to learn more”. “For me, if organisations are able to reduce the high turnover I would say there needs to be better pay, better working conditions, a reasonable workplace, all the things that can go on to making for a better work environment for the staff is gonna help, I don’t think that’s going particularly well in the services as far as I can see it”.(P. 92) |
| Finding | Good supervision and support- Participants who received support and supervision were happy with the help they received. (U) |
| Illustration | “The supervision helped, we were afforded two hours a month with the person of our choice, they had to be qualified.” (P. 94) |
| Finding | Good relationship with other health professionals- Some participants had good relationships with other health professionals, giving them a sense of belonging and acceptance. (U) |
| Illustration | “I found my nurse particularly helpful because they have a lot of experience and they have been though really horrible experiences themselves. A lot of the time they know exactly what you have had to deal with in terms of behaviour so they are like a really valuable resource”. “The psychiatric district nurses that we were involved with, we were all on the same page, we all knew how hard this job was and we knew that by and large we were very underappreciated but we all had the same focus and that was making sure these people were staying in one piece and when you’ve got that outlook on life and you really value that it has a very cohesive effect. There was a lot of solidarity between us and everyone else except psychiatrists but within our NGO there were some very qualified people, if anything there was a lot of admiration for the fact that we stuck with it in the face of all the of dangers and all the rest of it.” (87) |
| Finding | Difficulties in motivating clients to engage in activities/ complete daily chores (U) |
| Illustration | “It can just be physically draining and mentally draining when you are negotiating like that all day and they are not the only client, because we are a level four, we have clients who typically have a lot of behavioural issues and so it can just be that the battle that you were trying to fight yesterday you will fight again today. An example is trying to get people to do their chores because we have a roster for the housework and trying to get people to do that or trying to get people to cook on their rostered day, that’s just an on-going battle, and its one of the things that you can’t just give up because it is your role, we are trying to transition the people back in to the community so it’s an important part but it can be quite draining trying to obtain that.” “The interaction with clients can be challenging because of the reluctance or push back you might to call it, that’s probably there all the time. It may come and go but it’s usually there with somebody.” (P. 83) |
| Finding | The blurring of boundaries between informal and formal care work- Lack of professional boundary reinforcement by some support workers was reported to be challenging. (U) |
| Illustration | “…we were supposed to have professional boundaries but some staff would do things such as give the clients cigarettes, and then one time a client came in and asked another staff for a cigarette and they said no and he threw a cup at her and that was because other staff had been doing that so the client had the expectation that all staff were gonna give them cigarettes, then when you say no they get pissed off and that could have been really serious”. “A lot of support workers refused to believe that professional boundaries were useful…Some of the support workers would give their phone numbers out to clients and things like that which others of us would absolutely refuse to even consider, because that’s not even going to help anybody”. (P.81) |
| Finding | Inadequate safety support - Some participants reported that their colleagues had been in dangerous situations at work but did not receive support from the managers. (U) |
| Illustration | “In one incident a client came into the office to stab me. That was a rather unfortunate incident. I didn’t get any support from my organisation, they threatened to sack me if I went to the media about it. It was such a dangerous situation and a list of pho pars one after the other and I was very upset about it and I said to them if something is not done about it and someone isn’t held accountable for it I will go to the media.” “I ended up getting screamed at, yelled at by the client from a metre away and all you can do is just keep your calm and just pray to God they don’t hit you. If they hit you then you’ve a lot of grounds to do a lot. But until they hit you all you can do is stand your ground and that’s when I had a client who was actually in the hallway making sure that I didn’t get hurt. And it wasn’t until the third attempt when I went back and called my manager and said look this is what is happening, it’s not productive at all. It’s just aggravating the situation and she still said no I just had to keep doing it… when I rang back probably about four minutes later she said leave it because you guys are going to be late. So she was more worried about getting my client late as opposed to what had actually happened. So it was one of things where it is all about getting the job done, because safety can be put secondary”.(P. 102) |
| Finding | Negative experiences of working with other health professionals (U) |
| Illustration | “Clinical staff don’t do it intentionally but they treat you like a little kid, like you can’t think for yourself, they feel they have to make all of the decisions and they have to micro-manage you. And you feel like saying to them, excuse my language, but ‘fuck off’, of course that’s not what I say, but it’s actually quite offensive and some of these people are the same age as me, with less education than me”. “I never had any trouble with people not takings their meds. Although one time a woman took the wrong meds, it was my job to watch her take her medication, and we were talking, as I was still new and trying to build a rapport with her, and she took the wrong dose of medication. I felt really awful and I had to take her to the hospital, so med support is actually a really big responsibility, if they take the wrong medication it can be really serious, life threatening even. When I called the clinical team to tell them what had happened they made me feel really guilty and that I was a really stupid and made me feel terrible about it”. (P. 88) |
| Finding | Lack of support and guidance from the management (U) |
| Illustration | “As soon as you do something wrong, they come down so hard on you instead of supporting and training you. I mean that in all respect because I have had some really nice chats with the managers as well, but in general I don’t have a very supportive manager. How can they expect us to be recovery focussed if they are not treating staff in the same manner. It’s counter recovery.” “My manager was the one that made things very complicated. She had a very authoritarian way of dealing with things…we had fortnightly staff meetings…in all honesty the staff meetings felt like a telling off. ‘Who is she going to pick on now?’ So they weren’t very productive”. (P. 106) |
| Finding | Organisational hierarchy- Some participants reported feeling they were at the bottom level of the corporate order. (U) |
| Illustration | “So you hope that your manager would know that even though they have to deal with the upper management side of things, they are the person who can make a huge difference to you, to your work environment. Because you’re at the bottom of the line. You’re the one dealing with all the dirty work.” (P. 107-108) |
| Finding | Reliance on previous training - Some participants reported that they had to rely on training and skills they had received from their previous work. (U) |
| Illustration | “I had the benefit of voluntary work training, which was really useful and I applied a lot of the skills I learnt at previous voluntary work to this job. I noticed some of my other coworkers hadn’t had this same training and they would have really benefited from something like that. At my previous voluntary work they trained you in counselling skills which was listening, reflecting, which takes you out of the ‘trying to rescue the person role,’ which a few of the support workers had that sort of mentality, that these were people that they were going to go in and rescue, they knew exactly what the clients needed and would boss them around and stuff like that.” (P. 97) |
| Finding | Lack of induction - Some participants reported not receiving full induction or training before starting their work. They said that being aware of their professional boundaries was an essential aspect of their role. However, they did not receive training in this area until eight months into their role. (U) |
| Illustration | “And a lot of the clients as well have learned behaviours, you almost want to say emotional manipulation, but it’s not intentional it’s just the way they are and have learned to react to the people that come to help them so that they can get more help out of them and the other people in their lives, and not being aware of that and not being aware of your boundaries you do just become part of that, especially prior to the training I received about professional boundaries”. (P. 97) |
| Finding | Lack of or inadequate formal supervision (U) |
| Illustration | “We are a bit lacking in supervision with this particular company, I haven’t had any supervision. I have been with this company for about fourteen or fifteen months and I haven’t had any supervision there at this stage”. (P. 94) “The general support, such as supervision, was really poor, especially comparing this with previous voluntary phone counselling work I had done, the level of supervision we had for that was incredible, whereas supervision in mental health support work, it was kind of more like a training, it felt like the person running it just wanted to teach their thing, as opposed to working through any issues that you personally might be experiencing”. (P. 95) |
| Finding | Reliance on informal supervision from colleagues, family, and friends- Participants who did not receive formal supervision reported discussing work-related issues with colleagues or family and friends. (U) |
| Illustration | “The level of supervision we were receiving did not feel like it was adequate for the work we were carrying out with clients, there were certainly times where I would notice there were things I had done with clients that didn’t help them at all and then afterward, after talking about it with other people, I could see that if I had had the right support around this, as I was going through it, then it wouldn’t have been the issue that it was. So I had to talk to my co-workers or people outside of the organisation about the issues I was having with clients”. (P. 95) |
| Finding | Challenges related to finance allocation- Participants reported that non-government organisations focused on money and keeping the services running within tight budgets. (U) |
| Illustration | “I reckon its money focussed, that’s the only reason why the cuts and that were made. I think the clients are there to attract that budget that they have got to run the company, that’s what I think”. “Instead of being driven by health outcomes, primarily from what I understand it’s about money, it’s about the economics….I respect my colleagues and my managers and staff responsible for managing the budgets because they have had to make some difficult decisions around the level of staffing and service we provide, but they have managed to keep us afloat, as far as I understand we are in a viable position”. ( 111- 112) |
| Finding | Good relationships with colleagues- A good relationship with colleagues is beneficial. Colleagues are helpful for informal peer supervision, especially when there is inadequate individual supervision. (U) |
| Illustration | “So it is really helpful when you have got another staff member on, or doing activities together outside of work with co-workers, because they know what you go through. Not many other people know what the job is actually like”. “My co-workers were good to talk to and to vent to, as we all understood the frustrating things we were going through” (P. 86) |
| Finding | Implementation of recovery principles -Participants reported that recovery philosophy was mentioned in the visions and values of the organisations but not implemented in care delivery to clients or with the organisation's treatment of staff. (U) |
| Illustration | “Its not a role that I would say is recovery focussed, I don’t think we are practising recovery focused principles, I think we are more carers and so that’s what’s we are doing, caring for people day after day. The organisation doesn’t promote recovery at all, they have it in their visions and values and that’s it”. (P. 90) |
| Finding | Limited training- Some participants reported that the available training was either not enough, very bland, too generalised, not well implemented, and did not include the broad range of skills required to conduct support work effectively. (U) |
| Illustration | “There was some training provided but the time constraints meant the training was hard to access and also the training wasn’t really thought through very well. For example the training about keeping yourself safe, which is pretty basic when working with people out in the community who are psychotic, but that would have taken a horrible incident or a murder for example to actually do something about it and I think that hasn’t happened yet not with a mental health professional yet anyway.” (P. 96) |
| Finding | Lack of performance feedback- Some of the participants reported that they did not receive enough feedback from their supervisors about their work performance. (U) |
| Illustration | “No feedback is one of the other things I really noticed, you just go in to visit your client, fill in your paper work, nobody checks it, nobody does anything, then you just kind of catch up with your supervisor and let them know how your going in terms of how close you are to finishing with your client and that’s really it, if you need to change the hours with them, nothing about the content of what’s actually going on with clients” “There was no guidance around the decisions I was making when working with clients, I was very much working on a trial and error basis”. (P. 100) |
| Finding | Limited resources- Participants reported that the non-government organisations did not receive sufficient funding, resulting in limited availability of support work resources. (U) |
| Illustration | “…its sort of a typical behaviour with NGOs, were not well known for running really good services, were not really well known for recovery focused practises. It’s like even the unions said to me there a few weeks ago, it’s typical NGOs, they are underfunded from the DHB to do a job that the DHB once did themselves, that cost a lot more money so now they have got a real small budget to work with and that affects the quality of the managers that they have and you end up with a shoddy service.” “I found it challenging that mental health is a really underresourced area in general. And you can feel it just by the lack of resources you have to work with. Like day activities for the guys. It’s really hard you have to think outside the box. There’s not much for them to do”. (P. 110) |
| Finding | Qualification limitation- Some participants reported that the Certificate in Mental Health and Addictions, which was the core training for support work, was limiting both in terms of the course content and ability to lead to job progression or pay increases. (U) |
| Illustration | “…it’s sort of assumed or decided that any further knowledge is in the view of a registered nurse, if you want to know more or be more professionalised in your understanding then you need to study to be a registered nurse because only RNs are allowed to have that knowledge, and that’s the attitude, rather than encouraging people to learn as much as possible.” (P. 98) |
| Finding | Unrealistic goal-setting approach - participants reported that it was challenging to work on recovery because some of the clients were so unwell. (U) |
| Illustration | “A lot of the time the goal setting just went out the window, a lot of what you used to have to write down in the report was fabricated because that was what they needed to hear. We discussed this, we did that, when in fact the client didn’t even know what day of the week it was or what his name was. So very unrealistic a lot of it.” “The organisation did have a goal setting approach but it was very badly designed for mental health… You can’t tell a 53- year-old guy what are your dreams and aspirations? So you need to say ‘hey I know it is difficult to get out of bed, but do you know you have got eight hours that are really functional and you’ve got an unlimited car, and I can help so what do you like doing?’ It’s more of a realistic approach. So the company had a really good goal setting approach but it was not applicable to mental health”. (P. 89) |
| Finding | Lack of support, supervision, and appropriate training - Mental health support work was reportedly emotionally draining and required a lot of support. However, participants said that they did not get sufficient support to deal with the issues that came up at work. (U) |
| Illustration | “That would be one of the other things that stopped me from working there forever as well, the emotional side of the work, it is just too much for a job, you couldn’t always just leave the emotional aspects of the work at work, whereas in a similar voluntary role I did, you would deal with similar things but you could just leave it there, but I think that was because of the excellent supervision and training”. (P. 92 - 93) |
| Study: The contribution of the mental health support worker to the mental health services in New Zealand: An appreciative inquiry approach | |
| Finding | Personal gratitude- Genuine gratitude significantly affects Support workers' role. (U) |
| Illustration | "When you get a client that says thank you, when they are genuinely thankful. It has probably happened twice in the last seven months. I remember each of those days vividly and when you are having a tough time at work, that is what you think of, that time when that person told you that you are making a difference in their life." (P. 155) |
| Finding | Autonomy- Being valued and able to work autonomously is seen as a facilitator of support work role. (C) |
| Illustration | "We work under the supervision of a case manager, I was very fortunate that the case manager allowed me a lot of autonomy." (P. 188) |
| Finding | Clinicians' workload- Clinicians were perceived to be very busy and thus took a long time to respond to the support workers' concerns. (U) |
| Illustration | "They are just so flat out and you have to wait a month and a half to get an appointment and then on that day the doctor will be sick. They just always seem to be running. It seems like that. So if you ring a clinical worker because you have a concern, quite a few won’t get back for four or five days but I know they work four days on, three off. But it seems like I am always chasing them. And I think that if I have an issue or a concern with a client overall, eventually they will get back and they usually are really responsive in a positive way when they do but yeah, it’s short and sharp until next time." (P.162) |
| Finding | Cultural understanding of mental health - Pasifika people's understanding of mental illness differs from how it is described in Western medicine. As a result, they are reluctant to be seen by mental health support workers. (U) |
| Illustration | " Sometimes they don’t let you into their house. We only walk away and then we try again. Sometimes the doctors will go in first, like the Crisis Team and my work as a support worker, will help to fill the gap with the families because a lot of pacific islanders, Cook Islanders, they don’t believe in doctors and they don’t believe they have the mental illness. Where I come from they call mental illness, hearing voice is the ancestors telling you things." ( P. 136) |
| Finding | stratification of hierarchies- Support workers reported stratification between the various health professionals as well as within the mental health support work. Peer support workers were perceived to have the luxury of time as they had a small case load while community support workers were considered to be at a higher level than residential support workers. (U) |
| Illustration | "it was a little bit elitist, if you were a community support worker you were better that a residential support worker. The thing about our team is that we didn’t have the traditional clashing conflict we would ring them directly [clinical team] and they would come. It’s being better valued and being made to feel valued by the clinical services." (P. 187) |
| Finding | Acknowledgement of mental health support workers- Being accepted as part of the team and being valued for the skills and experiences that mental health support workers bring to the team is viewed as a facilitator for their role. (U) |
| Illustration | "I think as a whole, it is only as a team that we move forward. Like if you try and think of the things you do yourself, you are not going to go anywhere in a hurry, it is teamwork that makes a difference, not individual work." "Yes. Like the team has their own specific roles they do with medication that makes a difference, social worker and also ourselves we have our own specific roles that make a difference but bringing those together and it’s a nice wee combination for the person. Because everyone is different so you are tailoring different needs to different individuals." ( P. 180) |
| Finding | Professional boundaries- Support workers have long-term relationships with consumers, allowing them to know some aspects of consumers' lives that may be invisible to other health professionals. However, their opinion related to consumer recovery is usually ignored by health professionals. (U) |
| Illustration | "Yes different like that. And because they have been in this field for a long time they need that support which support workers can give. There are professional boundaries the doctors; they don’t want to take our comments as such. For the assessment even, there has been the increase in the medications for two or three of my clients because there are more medications, we have come to know that there are changes in the behaviour of clients, not always positive ones but because of some things and the medications. So the doctor says that because this has happened,but as staff, we should be asked how is it, is it because of some problems inside them, family issues or something or is it about their job." (P. 186) |
| Finding | Ability to work holistically- The support workers' role is flexible as it is not confined by the rigidity of regulations and legislation associated with other health professionals. This flexibility allows the support workers to get close to the feelings of the consumer. (U) |
| Illustration | "In my experience so far, I would say I find it quite hard sometimes to work with clinical because we work holistically, so we deal with the feelings and we see clients on a weekly basis so we have the one to one time and we get the whole picture, the whole holistic picture and we deal with their feelings and the stuff that goes on for them week to week. And with clinical they have to be very rigid and get their job done in that specific time, so they don’t go on gut feelings the way that we do and they see that client in a window." ( P.184) |
| Finding | Lack of collaboration between the clinical team and the mental health support workers- Participants reported difficulties related to poor communication and lack of collaboration between the clinical team and mental health support workers. (U) |
| Illustration | Yeah, so you wouldn’t know if there were any medication changes, you wouldn’t know what was going on, so you know communication again. "Communication breakdown in the community setting was huge. Then it comes down to client rights doesn’t it? That’s where the contact with the clinical team for letting you know, but not necessarily, they do eventually but not always straight away, it might be a few days. It’s just typical, normal. That’s what I mean as a support worker you have no control." (P. 178) |
| Finding | Educational pathway for mental health support workers- Challenges related to support work entry qualification were raised by the participants. Level 4 ( sub-degree level) qualification was viewed as an appropriate entry level with possibilities of specialisation within the same level. (U) |
| Illustration | "I don’t want to over-emphasize the qualification as opposed to losing what we have now, it’s a balance. I think the diploma sits quite well at the moment because a lot of it is on the things we are doing, it’s everyday stuff that we do. It depends on where you are taking it, with the bachelor, what is the area that you are taking it into. Is it more of the managerial type roles or running a service? At this stage I don’t think you need to be over-educated on the ground floor, there will come a time I suppose, the authorities will probably call for it, we have a degree in OT [occupational therapy], we have a degree in social work, we have a degree in nursing, so obviously if support work stays around then it will be the next step as well to work in the area." "I would like to see more streams in support work, more areas that we can move into, perhaps the work environment where there could be a role for support workers, work areas like that. There are a whole lot of levels where support workers could move into and a lot of employment opportunities." (P. 200) |
| Finding | Low pay- Support workers expressed the need to be recognised and compensated appropriately in terms of salary. (U) |
| Illustration | "We are very low paid for this because even if we are on holiday, we always think of work. We think we should be paid like a social worker. They are paid high and we are paid low. We are doing more than them. Only thing is they are social workers and we are support workers, but we are doing the same thing. We are only paid sixteen something dollars an hour. When you do something good for people who are really suffering, you will have good deeds for generations to come. If it’s a referral for us, they come here. We get referrals from them and we also get to see the doctors and nurses because we are taking clients there. We have meetings there. So we often go there, so we often get to see them. Sometimes there is a difference of opinion. They think that support workers that we don’t do anything, but we do heaps of things. I feel that way, but I don’t know how others feel. Sometimes I feel really frustrated, and say why are we paid for less and why we have to take more. Sometimes I feel frustrated and tell my colleagues." (P. 204) |
| Study: The experiences of support workers of people with schizophrenia in Winnipeg, Manitoba | |
| Finding | Support plan as a guide for care- Support plans were seen as a guide that provided the support workers with information regarding the clients' support needs and any other relevant information related to a client. (U) |
| Illustration | "With the advantage, I will say it serves as a guide to both new and old staff. It updates us on the current detailed needs of the clients for effective support work. With the support plan, you get the chance to know what is needed for the client and help to make our work easy." (P. 41) "If any staff [both casual and permanent] enters a home, the first thing that guides them as to how to deal with the client starting from his/her name, allergies, and his/her daily routine is the support plan." (P. 46) |
| Finding | Aggressive behaviour- Participants reported that they experienced aggression from some of their clients. (U) |
| Illustration | "Also, the support plan doesn’t present any form of security or insurance for staff when they are attacked or abused. I have personally encountered several attacks from aggressive clients without any form of compensation or insurance. Due to this, I feel the support work can sometimes be risky." (P. 41) “I have witnessed several cases of abuse including racial abuse, sexual abuse, and harassment from clients which sometimes makes me lose interest in this job.” (P. 59) |
| Finding | Limitations of support plans- Participants reported that they were supposed to provide support as stipulated in the support plans. The support plans did not allow the support workers to use their judgment to make adjustments or changes to the support provided. The clients were also not given choices regarding their care. (U) |
| Illustration | Sometimes you would wish you can do more or allow something for a client then we are restricted, and we are only supposed to do what is stated in the support plan. So even if you’d wish to do more or you’d wish to take out some things from the plan, you are not allowed to do so because that will be like you going against the plan." (P. 42) "The people we support are adults. They are mature and can decide on most parts of their daily activities. But it feels bad when you are not allowed to do what you feel like doing at a certain moment because you are in a care home. If I want to go out and can only do it at a time the support plan says, then it feels very unfair." (P. 46) |
| Finding | Support workers' input to clients' support plan - Participants stated that their input and suggestions related to clients' support needs were not included in support plans. (U) |
| Illustration | "Indeed, I cannot count the number of times I’ve made suggestions to improve the support plan but [they] have all been ignored or not taken..." (P. 41) "I have been in several end-of-month meetings where management will ask us about our challenges and recommendations for the people we support. In these meetings, many challenges are shared by my colleague workers and myself but hardly are any of these suggestions put into use or made available in the support plan. I think the meetings have just become mere formalities but not to take what we suggest into use." (P. 60) |
| Study: Support workers in community mental health teams for older people: roles, boundaries, supervision and training | |
| Finding | Limited training opportunities- Support workers reported limited training opportunities for their role. (C) |
| Illustration | “When there are certain trainings like cognitive behavioural therapies and they say “oh no, it’s not for you, it’s for professionals”. Then I get hurt, and I get angry because it’s almost suggesting that I am probably not capable of learning that, or perhaps within my role it’s not really necessary, which is bumpf.” (P. 15) |
| Finding | Inadequate support- Support workers reported insufficient support and supervision in the community setting compared to a hospital setting. (U) |
| Illustration | “On the ward you are supervised by a qualified nurse. In the community you are out there on your own. Whether they say: “well you have got a CPN at the end of the phone, you have only go to ring, you know”, there is not always somebody available." (P. 14) |
| Understanding the Experiences of Mental Health Support Workers in Independent/Supported Living Facilities: A Thematic Analysis | |
| Finding | Inadequate training- MHSWs perceived their training to be too theoretical and generic and was not always relevant to the needs of the service users they supported. (U) |
| Illustration | ‘Yeah, it does help you, but at the same time it’s more general than direct to their needs or to yours […] when you do the training is not designated to the person, so it’s more in general. It’s not for the person you support and not everyone is in the same frame. |
| Finding | Dealing with verbal abuse- Most participants reported experiencing verbal abuse which had negative impact on their well being. They reported that they try to manage these challenges through rationalisation of a service users behaviour based on their diagnosis, remaining professional and not taking things personal. (U) |
| Illustration | ‘You don’t take it personally, but it could still be stressful in the moment when you’re being shouted at by someone like it’s always a bit challenging [….] I mean it’s hard as I said, to shout at you or tell you that they hate you, that they never want to work with you again [….] It’s nothing to do with me, really.’ |
| Finding | Role confusion- MHSWs perceived their role to be vague and confusing with some reporting that their actual job responsibilities were different from what they anticipated based on their job description. (U) |
| Illustration | ‘So, on paper, it is very clear, so as a support worker you’ve got, what they expect from you, but what they expect from you on your day-to-day job, is two different things.’ |
| Finding | Support from managers- Most participants felt supported by their managers and they could reach out to them if they were having issues. |
| Illustration | ‘At the start of the year, I had a bit of a breakdown myself. Then I had about five weeks off work. And they were brilliant they would always be ringing me [….] Yeah, even though I was not at work, I never felt like I was on my own [….] Or like the seniors in the managers were ringing me or sending me an email just saying like just checking in.’ |
